# Deep Riemannian Networks for end-to-end EEG decoding

**DOI:** 10.1162/imag_a_00511

**Published:** 2025-03-21

**Authors:** Daniel Wilson, Robin T. Schirrmeister, Lukas A. W. Gemein, Tonio Ball

**Affiliations:** Neuromedical A.I. Lab, Department of Neurosurgery, Medical Center—University of Freiburg, Faculty of Medicine, University of Freiburg, Freiburg, Germany; BrainLinks-BrainTools, IMBIT (Institute for Machine-Brain Interfacing Technology), University of Freiburg, Freiburg im Breisgau, Germany; Medical Physics, Department of Diagnostic and Interventional Radiology, Medical Center—University of Freiburg, Faculty of Medicine, University of Freiburg, Freiburg, Germany

**Keywords:** EEG, filterbank, Riemannian and Deep Learning

## Abstract

State-of-the-art performance in electroencephalography (EEG) decoding tasks is currently often achieved with either Deep-Learning (DL) or Riemannian-Geometry-based decoders (RBDs). Recently, there is growing interest in Deep Riemannian Networks (DRNs) possibly combining the advantages of both previous classes of methods. However, there are still a range of topics where additional insight is needed to pave the way for a more widespread application of DRNs in EEG. These include architecture design questions such as network size and end-to-end ability. How these factors affect model performance has not been explored. Additionally, it is not clear how the data within these networks are transformed, and whether this would correlate with traditional EEG decoding. Our study aims to lay the groundwork in the area of these topics through the analysis of DRNs for EEG with a wide range of hyperparameters. Networks were tested on five public EEG datasets and compared with state-of-the-art ConvNets. Here, we propose end-to-end EEG SPDNet (EE(G)-SPDNet), and we show that this wide, end-to-end DRN can outperform the ConvNets, and in doing so use physiologically plausible frequency regions. We also show that the end-to-end approach learns more complex filters than traditional bandpass filters targeting the classical alpha, beta, and gamma frequency bands of the EEG, and that performance can benefit from channel-specific filtering approaches. Additionally, architectural analysis revealed areas for further improvement due to the possible under utilisation of Riemannian specific information throughout the network. Our study, thus, shows how to design and train DRNs to infer task-related information from the raw EEG without the need of handcrafted filterbanks and highlights the potential of end-to-end DRNs such as EE(G)-SPDNet for high-performance EEG decoding.

## 1 Introduction

Optimizing the amount of information that can be extracted from brain signals such as the Electroencephalography (EEG) is crucial for Brain-Computer Interface (BCI) performance and thus for the development of viable BCI applications. The multivariate EEG data are inherently complex as task-relevant information can be found at multiple frequencies, as well as in the correlation structure of the signals across different electrodes. Also, the generative processes which give rise to the EEG signals are not fully understood. To handle such complex data, most current EEG-BCIs use Machine Learning (ML) for the analysis of the recorded brain signals.

Currently, the state-of-the-art in EEG-BCI decoding with ML is often achieved through two relatively distinct strategies that have been developed in parallel over the last decade, Deep-Learning (DL) (Lawhern et al., 2018;LeCun et al., 2015;Schirrmeister et al., 2017) and Riemannian-Geometry-based decoders (RBDs) (Barachant et al., 2013;Chevallier et al., 2022;Congedo et al., 2017;Lotte et al., 2018;Yger et al., 2017). RBDs employ concepts from Riemannian geometry to leverage inherent geometrical properties of the covariance matrix of the EEG (Barachant et al., 2013;Congedo et al., 2017;Lotte et al., 2018;Yger et al., 2017). The covariance matrix captures task-relevant spatial information in not only the variances of the signals from single electrodes, but also the covariance between electrode pairs. DL uses multi-layered artificial neural network models and training using backpropagation to learn to extract hierarchical structure from input data, which has led to state-of-the-art performance in a number of fields, including EEG decoding (Lawhern et al., 2018;LeCun et al., 2015;Schirrmeister et al., 2017). The network’s multilayered structure means that they are able to learn sequential and/or parallel data transformations which were not pre-defined manually. This has led to the development of*end-to-end*models for EEG decoding (such as those bySchirrmeister et al. (2017)) that can be fed raw or minimally preprocessed data. An advantage of such end-to-end analyses is that processing steps such as data whitening or feature extraction are implicitly learned and optimised jointly with the classification during model training. Therefore, while DL can be viewed as a more general classification model, in principle suitable to extract and use arbitrary learned EEG features, RBDs are specialised to optimally leverage the information contained in the EEG covariance structure.

In the last few years, the success of RBDs and DL on EEG decoding and on other domains has motivated a number of works that sought to combine these two methods, leading to various types of Deep Riemannian Networks (DRNs). Some are orientated towards manifold valued data (Chakraborty, 2020;Chakraborty et al., 2018;Pennec, 2020), such as the arrays produced by Diffusion Tensor Imaging (DTI) but not EEG. Others are designed to operate on symmetric-positive definite (SPD) matrices (the category of matrix to which the covariance matrix belongs) (Acharya et al., 2018;Brooks et al., 2019a;Dong et al., 2017;Huang & Gool, 2016;Liu et al., 2019;Suh & Kim, 2021;Sukthanker et al., 2021;Yu & Salzmann, 2017). While some of these methods are demonstrated on facial/image recognition datasets (Acharya et al., 2018;Brooks et al., 2019a;Dong et al., 2017;Huang & Gool, 2016;Liu et al., 2019;Yu & Salzmann, 2017), some have also been demonstrated with EEG (Ju & Guan, 2022;Kobler et al., 2022;Liu et al., 2019;Majidov & Whangbo, 2019;Suh & Kim, 2021;Yang et al., 2020;Zhang & Etemad, 2020). One particular model of note is the SPDNet (Huang & Gool, 2016) (demonstrated on action, emotion, and facial recognition data) which was designed to mimic some of the functions of a convolutional network, but entirely on SPD data.Table 1summarises the literature concerning Deep Riemannian Networks for EEG. Of the literature seen inTable 1, SPDNet-like networks are the most widely used DRNs, with several of the cited works using various combinations of its layers within their architecture (Hajinoroozi et al., 2017;Ju & Guan, 2022;Kobler et al., 2022;Liu et al., 2019;Suh & Kim, 2021). Since these networks excel at extracting relevant spatial-spectral information from the EEG, the majority of these models (Ju & Guan, 2022;Kobler et al., 2022;Liu et al., 2019;Majidov & Whangbo, 2019;Suh & Kim, 2021;Yang et al., 2020;Zhang & Etemad, 2020) were demonstrated on motor EEG data—where the spectral power at different electrodes is known to be a prominent source of information. The differing architectures for these works suggests there to be no clear choice for SPDNet architecture on EEG, including the filterbanking applied pre-SPDNet.

**Table 1.** Deep Riemannian Networks for EEG.

Authors	$N_{f}$	Filter specificity	Learnable or pre-defined
Hajinoroozi et al. (2017)	1	Channel Independent	Pre-defined
Liu et al. (2019)	1	Channel Independent	Pre-defined
Majidov and Whangbo (2019)	4	Channel Independent	Pre-defined
Yang et al. (2020)	43	Channel Independent	Pre-defined
Zhang and Etemad (2020)	50	Channel Independent	Pre-defined
Suh and Kim (2021)	1	Channel Independent	Pre-defined
Ju and Guan (2022)	9	Channel Independent	Pre-defined
Kobler et al. (2022)	4	Channel Independent	Learnable
**EEGSPDNet** (proposed)	8	Channel Specific	Learnable

Table showing previous DRNs for EEG along with filterbank architectures as they would be described in this study. For comparison, our best-performing proposed model is also included. All models, with the exception of the work byHajinoroozi et al. (2017), were evaluated on motor data.$N_{f}$denotes the number of filters in the filterbank (for channel independent filters) or the number of filters per electrode (for channel-specific filters).

**Table 2.** Final evaluation set accuracies.

Dataset	**EEGSPDNet**	**FBSPDNet**	Deep4Net	EEGNetv4	ShFBCSPNet	TSMNet
BNCI2014001	74.4	66.5	63.0	64.3	72.5	74.9
BNCI2014004	77.8	74.8	79.6	81.4	78.8	75.2
Lee2019 MI	67.9	62.3	59.5	62.2	66.7	65.9
Schirrmeister2017	95.2	92.8	88.1	91.2	94.0	94.5
Shin 2017A	74.0	66.3	58.9	56.1	65.5	74.9

Left-most column shows the dataset name. Each subsequent column shows the final evaluation set classification accuracy achieved by that model. Values have been rounded to 3 significant figures. For spacing reasons the following model names have been abbreviated, in comparison toFigure 3, which is showing the same underlying data: EE(G)-SPDNet ChSpec>EE(G)-SPDNet, FBSPDNet ChInd>FBSPDNet and ShallowFBCSPNet>ShFBCSPNet. Precise details on the models and datasets can be found inSection 2.3andSection 2.4for EE(G)-SPDNet and FBSPDNet,Section 2.8for the comparison models, andSection 2.9for datasets.

Since a variety of subject-specific frequency bands may contain task-relevant information, EEG decoding pipelines regularly feature a filterbanking step designed to separate the incoming signal into multiple frequency bands (Akter et al., 2020;Ang et al., 2012;Belwafi et al., 2014,^2018^;Islam et al., 2018;Ju & Guan, 2022;Kobler et al., 2022;Majidov & Whangbo, 2019;Mane et al., 2021;Mousavi & de Sa, 2019;Schirrmeister et al., 2017;Thomas et al., 2008,^2009^;Wu et al., 2021;Yang et al., 2020;Zhang & Etemad, 2020). For methods like RBDs that use variance information, this filterbanking approach is a logical step: Different frequency bands are well-known to reflect different aspects of the brain’s functional connectivity, and hence the covariance structure of the recorded EEG signals is strongly affected by the frequency composition of the input signal. While filtering or filterbanking is a ubiquitous step for EEG decoding, the literature summarised inTable 1shows that for DRNs there is no clear-cut filterbanking method or architecture. The filterbank can either be constructed from a set of predefined candidate filters or by freely learning the filters (e.g., learning the cut-offs, or convolutional kernel). We will call the first construction method pre-defined filterbanks and the second method learnable filterbanks.

Pre-defined filterbanks have been used in many related areas, including Common Spatial Patterns (CSP) (Ang et al., 2012;Belwafi et al., 2018;Thomas et al., 2008,^2009^), RBDs (Islam et al., 2018;Wu et al., 2021), and DRNs (Ju & Guan, 2022;Yang et al., 2020;Zhang & Etemad, 2020) and consist of an array of specific filters. Despite being explicitly defined, these filterbanks are frequently large and the filters are often searched through in order to reduce feature dimension (Ang et al., 2012;Belwafi et al., 2018;Islam et al., 2018;Thomas et al., 2008,^2009^;Wu et al., 2021), although some models use all filters and do not explicitly reduce feature dimension at this stage (Ju & Guan, 2022;Mane et al., 2021;Yang et al., 2020;Zhang & Etemad, 2020). Search metrics vary, but all are intended as a proxy for class separability, be it the Fisher information ratio (Thomas et al., 2008,^2009^), mutual information (Islam et al., 2018), or a statistical test (Wu et al., 2021). Due to their explicit nature, they are easy to interpret and implement, but also require*hand-crafting*which may preclude some level of domain knowledge. Making the filterbank learnable eliminates this limitation and enables true end-to-end model training.

Learnable filterbanks are those that are flexible and not limited to a pre-defined set of filters but instead search a much larger space as part of an optimisation process, and they have been generally explored within the confines of DL (Kobler et al., 2022;Li et al., 2019;Mousavi & de Sa, 2019;Schirrmeister et al., 2017). These deep models have multiple parallel convolutional layers which are intended to mimic the effects of bandpass filtering on EEG, first introduced bySchirrmeister et al. (2017). However, the convolutional layer is not guaranteed to learn a strict bandpass filter, through random initialisation and following the optimisation it may also learn a notch, low-pass, high-pass filter or even a multi-band filter. The process of optimising the convolutional layer is essentially searching the band-space with the backpropagated loss of the network serving as the search metric, which, it can be argued, is a more direct measurement of class separability. This search process is not limited to a pre-defined set of bands/band-types and is inherently less granular and also more adaptable to datasets with a wider range of frequencies (such as those designed to measure frequencies in the high-gamma region). Learnable filterbanks remain underutilised in EEG decoding, particularly in DRNs.

As can be seen fromTable 1, there is a relatively small amount of work exploring DRNs in the context of EEG (Hajinoroozi et al., 2017;Ju & Guan, 2022;Kobler et al., 2022;Liu et al., 2019;Majidov & Whangbo, 2019;Suh & Kim, 2021;Yang et al., 2020;Zhang & Etemad, 2020). Furthermore, a number of these models have Riemannian transforms prepended onto standard deep networks (Majidov & Whangbo, 2019;Yang et al., 2020;Zhang & Etemad, 2020), as opposed to being based on the SPDNet (Huang & Gool, 2016), where the data are manipulated in the Riemannian space through many layers of the network (Hajinoroozi et al., 2017;Ju & Guan, 2022;Kobler et al., 2022;Liu et al., 2019;Suh & Kim, 2021). Moreover, even fewer of these methods used a multispectral approach (Ju & Guan, 2022;Kobler et al., 2022;Majidov & Whangbo, 2019;Yang et al., 2020;Zhang & Etemad, 2020) and most used a pre-defined filterbank (Ju & Guan, 2022;Majidov & Whangbo, 2019;Yang et al., 2020;Zhang & Etemad, 2020) with the exception of the domain adaptation focused TSMNet (Kobler et al., 2022) (seeTable 1). This is despite the theoretical advantages that learnable filterbanks possess.

Therefore, how DRNs can be best equipped with learnable filterbanks and how to gain neurophysiological insights from the learned filterbanks remains unclear. The learning of the filterbank can be achieved in an end-to-end manner, with parallel convolutions, or with a black box optimiser using typical bandpass filters, and it is not known which method is superior. In addition to this, the size of the filterbank and its channel specificity can also be altered. Furthermore, how much DRNs use well-known regions in the EEG (e.g., alpha, beta, and high-gamma for motor movement) has gained only limited insight (Ju & Guan, 2022;Kobler et al., 2022). It is not yet clear how these factors will affect performance, or whether Deep Riemannian Networks (DRNs) with learnable filterbanks can compete with state-of-the-art networks.

Therefore, we designed a study to address these questions, wherein we propose and systematically compare two SPDNet-based DRNs with learnable filterbanks for EEG, the end-to-end EEG SPDNet (EE(G)-SPDNet), and the Filterbank Optimised EEG SPDNet (FBSPDNet). EE(G)-SPDNet is a second-order convolutional neural network (Brooks et al., 2019b;Li et al., 2020;Yu & Salzmann, 2017), comprising a convolutional layer designed to mimic filterbanking, followed by a covariance pooling layer and then an SPDNet. FBSPDNet uses a black-box optimiser to search for an optimal array of bandpass filters. The post-filterbank data are then used to generate the covariance matrices that are passed to the SPDNet. These two models embody distinct methodologies for deep-RBDs utilising learned filterbanks. They are systematically evaluated and contrasted across various parameters, including filter type, filterbank channel specificity, and filterbank size, among others.

The design of the EE(G)-SPDNet allows for the learning of a custom filterbank from the input data while also being able to use Riemannian geometry to process data on the SPD manifold. The EE(G)-SPDNet can be separated into two stages, a convolutional stage followed by a standard SPDNet. The SPDNet stage reduces the size of the input SPD matrix, layer-by-layer, while preserving class discriminability before applying a Riemannian transform to whiten the data prior to classification. The convolutional layer filters the EEG signals and, over multiple iterations, becomes optimised to select the ideal frequencies for the covariance matrix. This removes the need for explicit filterbank architecture design, offering a more precise search of the frequency space. This precision can be enhanced as the convolutional filters can be optimised for each individual input channel, creating a channel-specific filtering (ChSpec) filterbank, something that could be cumbersome to design manually. Furthermore, the convolutional layer allows EE(G)-SPDNet to be flexible in adapting to datasets of different sampling rates (and therefore different frequency ranges) since the filterbank architecture does not have to be redesigned, nor the data low-pass filtered to accommodate the filterbank. Overall, the convolutional layer prepended to the SPDNet allows it to learn an optimal filterbank, as well as potentially create a filterbank composed of various filter types (notch, bandpass etc).

The FBSPDNet uses regular bandpass filters in conjunction with a Bayesian Optimiser (BO) to search the filterbank space, as opposed to the convolution in EE(G)-SPDNet—the SPDNet stage remains the same. At each step in the iterative search, a filterbank is constructed which is used to filter the data before creating the set of SPD matrices. The performance of the filterbank is evaluated via cross-validation with a lightweight proxy classifier on the aforementioned matrices. The matrices created from the highest scoring filterbank are then passed to the SPDNet. The FBSPDNet possesses almost all the previously mentioned advantages of the learnable filterbank approach such as channel-specific architecture and adaptability between datasets, and it is restricted only in terms of its filter type.

The bandpass filters used by FBSPDNet are more rigid in their approach to filtering when compared to the convolutional kernel, which may learn things other than a bandpass filter, but this also gives the FBSPDNet a smaller space to search. On the whole, FBSPDNet represents a model that is somewhere in between the EE(G)-SPDNet and the traditional filterbank approach, with the main difference to the traditional methods being that its array of bandpass filters are learned, rather than explicitly pre-defined. Additionally, we also test an EE(G)-SPDNet variant, where the initial convolutional layers have been swapped for Sinc layers, adapted from SincNet (Ravanelli & Bengio, 2019). This model variant helps bridge the gap between EE(G)-SPDNet and FBSPDNet, as it uses the same bandpass filtering as FBSPDNet, but the filterbank is optimised jointly with the rest of the network, as in EE(G)-SPDNet. Through the experiments performed in this study, we show that our models are capable of outperforming other state-of-the-art classifiers, namely the convolutional networks ShallowFBCSP, Deep4 (Schirrmeister et al., 2017), EEGNetv4 (Lawhern et al., 2018), and the concurrently developed DRN TSMNet (Kobler et al., 2022) with statistically significant improvements achieved by EE(G)-SPDNet against every model except TSMNet. Broadly, these results also demonstrate the effectiveness of DRNs (EE(G)-SPDNet, FBSPDNet, and TSMNet) against traditional convolutional networks (Deep4Net, ShallowFBCSPNet, and EEGNetv4). These improvements were shown on 5 public EEG motor datasets: Schirrmeister2017 (Schirrmeister et al., 2017), BNCI2014001 (Leeb et al., 2007), BNCI2014004 (Tangermann et al., 2012), Lee2019 MI (Lee et al., 2019), and Shin2017A (Shin et al., 2017).

In addition to analysing the performance of the different models, we also analysed some of the features learnt by the networks, and investigated the network’s layer-by-layer performance. We identified the frequencies that contribute the most to classifier performance by looking at the frequency gain caused by the trained filterbanks. We also visualised and tested separability of the data as it passes through the network in both Euclidean and Riemannian spaces.

## 2 Methods

### 2.1 Outline

The structure of our paper is as follows. The following methods section will cover: the necessary theoretical background (Section 2.2), details of EE(G)-SPDNet and FBSPDNet (Sections 2.3and2.4), network design choices common to both proposed models (Section 2.5specific SPD matrix operations (2.6)), methods used for analysis (Section 2.7), the datasets that were used (Section 2.9), the experimental procedure (Section 2.10), and the statistical methods used for presenting certain results (Section 2.11). After this, the results will be presented with figures, in a series of key findings (Section 3), before a discussion of the main points (Section 4) and the subsequent conclusion (Section 5).

### 2.2 Riemannian methods overview

Riemannian methods for EEG are usually applied on the sample covariance matrix^*^of the EEG electrodes:

$$C=\frac{1}{N_{t}-1}TT^{⊤}$$ (1)

where$T\;\in {\mathbb{R}}^{N_{e}\times N_{t}}$. For EEG,Twould represent a single trial of$N_{e}$electrodes and$N_{t}$samples.Equation 1is the equation for the Sample Covariance Matrix (SCM), which is, by construction, both symmetric and positive semi-definite (all its eigenvalues are greater than or equal to zero). Furthermore, if sufficient data are used ($N_{t}$>$N_{e}$) andThas full-rank (i.e., no interpolated channels), thenCwill be positive definite (all eigenvalues are greater than zero).

RBDs exploit geometric properties of the SPD manifold, on which the computed SCMs reside. They use tools from Riemannian geometry to make computations on the SPD manifold, which has helped them to achieve state-of-the-art performance. These computations rely on the ability to accurately measure distances on the curved, smooth SPD manifold, properties that arise from the non-linear but open condition of positive-definiteness. At any point of the SPD manifold, a vector space can be constructed, called the tangent space. The tangent space at a pointprepresents a locally Euclidean approximation of the neighbourhood aroundp. A metric on the manifold defines an inner product on each of these tangent spaces, and this inner product varies smoothly as we move from point to point. When equipped with such a metric, the SPD manifold becomes a Riemannian manifold. Geodesics represent the shortest distances between points on the manifold, however they may differ depending on the chosen metric—in a flat space, they are straight lines. In practice, the SPD matrices are mapped to the Euclidean tangent space using Riemannian geometry, preserving their structure from the SPD manifold (this is how the Riemannian Support-Vector Machine (rSVM) is implemented here, a Riemannian transform followed by SVM classification). For further information on the mathematics and details of Riemannian Geometry and its applications to EEG, we direct the reader toBarachant et al. (2013)andYger et al. (2017)and for some specific details on Riemannian metrics we suggestHuang et al. (2015)andArsigny et al. (2006).

This study employs the SPDNet, which was first introduced byHuang and Gool (2016), and is a deep network designed to “*non-linearly learn desirable SPD matrices on Riemannian manifolds*” (Huang & Gool, 2016). The SPDNet uses three SPD specific layers to process the input data. BiMap layers are used to reduce SPD feature dimension and enhance discriminability. For the$k^{th}$layer this is done to an input matrix$C_{k-1}$as follows:

$$C_{k}=f_{b}^{\left(k\right)}\left(C_{k-1},W_{k}\right)=W_{k}^{⊤}C_{k-1}W_{k}$$ (2)

where$W_{k}$is the learned weight matrix (must be constrained to a Stiefel manifold of dimension$d_{k-1}\times d_{k}$).

The ReEig layers aim to mimic the Rectified Linear Unit (ReLU) by preventing eigenvalues from becoming too small, (as the ReLU prevents negative values). The ReEig function is defined as follows:

$$C_{k}=f_{r}^{\left(k\right)}\left(C_{k-1}\right)=U_{k-1}max\left(\in \text{​}\;I,\Sigma_{k-1}\right)U_{k-1}^{⊤}$$ (3)

wherekis the layer index,Ian identity matrix, and∈is a threshold of rectification. An eigenvalue decomposition onCleads toUand eigenvaluesΣ. Themax()function in this case forces the elements of the diagonal matrixΣto be above the threshold value,∈. This prevents the matrices from getting to close to the singular boundary of the set of positive definite matrices of sizen,$S_{n}^{pd}$.

As inHuang and Gool (2016), the BiMap and ReEig layers are applied in pairs, with each successive BiMap reducing the size of the input matrix by half. The depth of the network is then defined by how many BiMap-ReEig pairs there are, denoted$N_{BiRe}$.

The final Riemannian layer is the LogEig layer which transforms the input data into Euclidean space using the Log-Euclidean Metric (LEM) (Arsigny et al., 2006;Huang & Gool, 2016). This means a matrix logarithm is applied to each matrix in the input batch. This is faster than applying the logarithmic map from the Affine Invariant Riemannian Metric (AIRM), and there is no need for determining a reference matrix. The speed difference means it is better suited to the deep learning pipeline, where it will be used for every epoch.

The LogEig function,$f_{l}$, is defined as follows:

$$C_{l}=f_{l}^{\left(k\right)}(C_{k-1})=log(C_{k-1})=U_{k-1}log(\Sigma_{k-1})U_{k-1}^{⊤}$$ (4)

where$C=U\Sigma U^{⊤}$is an eigenvalue decomposition andkis the layer index.

After this, the output can be passed to a regular fully connected layer for label prediction.

### 2.3 End-to-end EEG SPDNet

The EE(G)-SPDNet architecture, illustrated inFigure 1, incorporates two layers atop the SPDNet for end-to-end processing of EEG signals. A convolutional layer similar to that inSchirrmeister et al. (2017)temporally filters the input signals via convolution. The SCM pooling layer then creates SCMs from the convolved (i.e., filtered) signals and passes them to an SPDNet. This design allows joint optimisation of the convolutional filterbank with model training. Two types of filter have been used in this study, a regular convolutional filter and a sinc filter. Both filters filter the incoming signal in the frequency domain via convolution in the time domain, and both use the same length convolutional kernel. The sinc layer, adapted from an implementation of SincNet (Ravanelli & Bengio, 2019), generates the convolutional kernel from sinc functions, resulting in a bandpass filter frequency response. Its learned parameters are the low/high cutoff frequencies of the bandpass filter. Conversely the “regular” convolutional filter directly learns the convolutional kernel values. While the sinc layer is readily interpretable as a bandpass filter, the regular convolutional filter is less restricted, potentially learning sinc filters but also potentially learning more complex filter types.

**Fig. 1.**
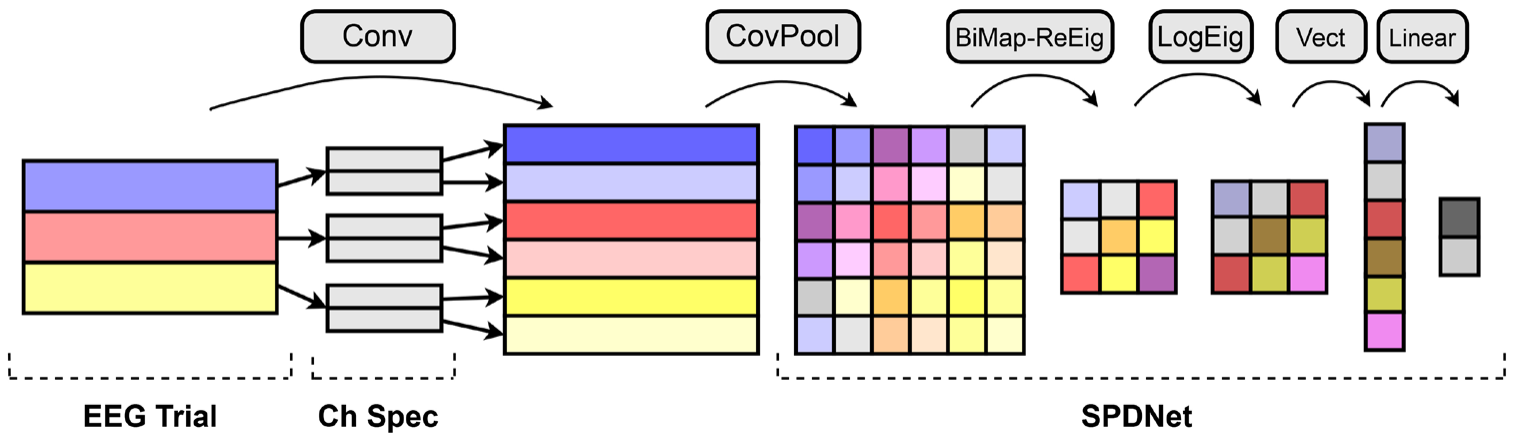
Diagram showing the architecture of the EE(G)-SPDNet. Input trials are convolved in a convolutional layer, which mimics bandpass filtering. An SCM pooling layer turns the convolved time series into sampled covariance matrices, which are SPD. These matrices are then passed into an SPDNet to produce class predictions. The EE(G)-SPDNet shown here has had certain layers removed to improve clarity; therefore, it should be noted that the EE(G)-SPDNet used for computations has 3 pairs of BiMap-ReEig layers, and that the BiMap and ReEig layers are separate layers. For more detail of the SPDNet components, see the work byHuang and Gool (2016).

### 2.4 Optimised filterbank EEG SPDNet

FBSPDNet combines a separate Filterbank Optimisation (FB-Opt) loop with covariance matrix generation, followed by a regular SPDNet. It employs a BO (although any black box optimisation algorithm could be used) to search the frequency space for the optimal filterbank, as shown inFigure 2. The optimiser iteratively adjusts filterbank parameters (bandpass filter cut-offs) to maximise cross-validation accuracy on the computed covariance matrices. To reduce computational burden, a lightweight proxy classifier (Support-Vector Machine (SVM) or Minimum Distance to Mean Classifier (MDM) with a Riemannian metric) is used instead of training/testing an SPDNet at each iteration. Both classifiers were tested with LEM and AIRM Riemannian metrics. These Riemannian-based classifiers (rSVM and Minimum Distance to Riemannian-Mean Classifier (rMDM)) have been previously used as RBDs for EEG (Barachant et al., 2013;Congedo et al., 2017,^2019^). In order to ensure fair comparisons with Sinc-EE(G)-SPDNet, the filterbank for FBSPDNet was constructed from static sinc-convolution layers.

**Fig. 2.**
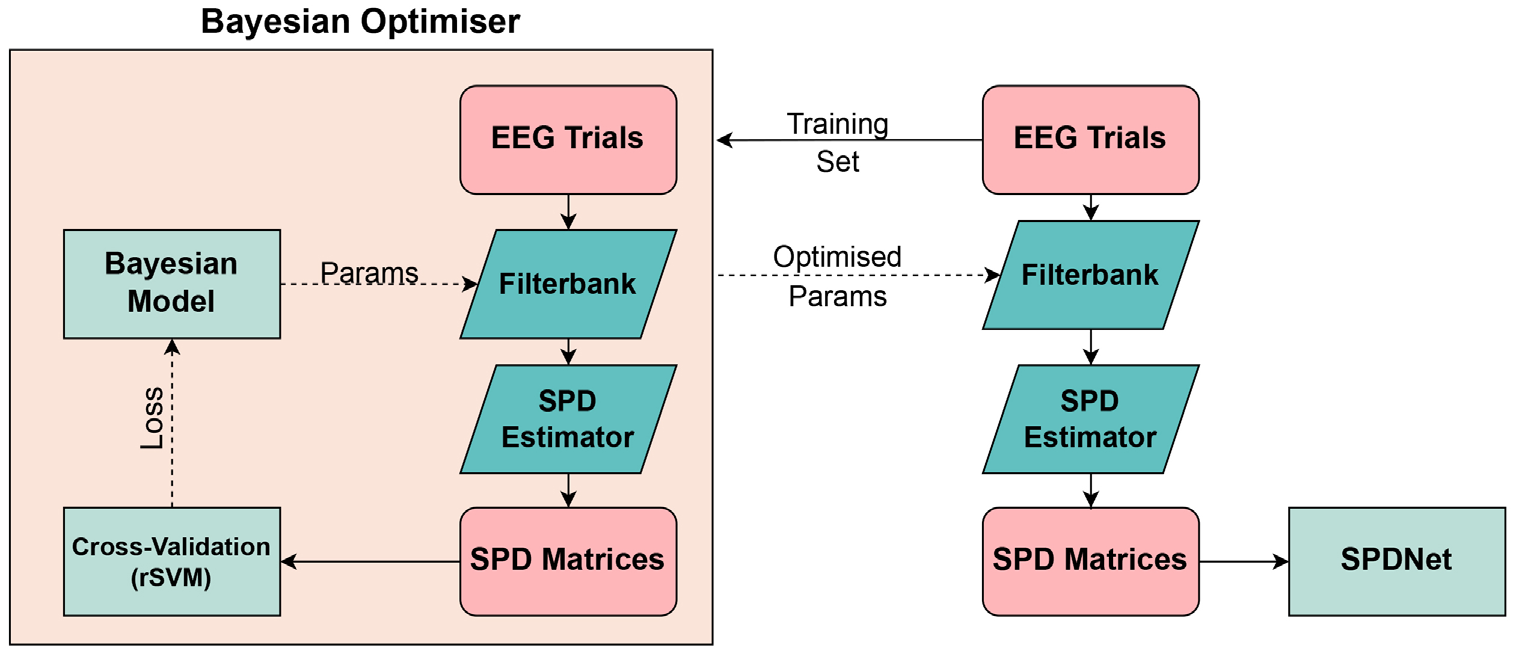
Diagram showing the architecture of the FBSPDNet. The training set is passed to a BO, where it is used to optimise the filterbank. The optimal filterbank is then used for the entire dataset. An SPD estimator (in this case the SCM) is used to create SPD matrices, which are passed to the SPDNet for classification.

This optimisation loop results in a set of easily interpretable filterbank parameters that are optimised to the data. The SPD matrices created from the optimised filterbank are passed to an SPDNet. This SPDNet is identical in architecture to the one in the EE(G)-SPDNet.

### 2.5 Network design choices

In the following section, we elaborate on some design choices specific to the SPDNet, but are applicable to FBSPDNet and EE(G)-SPDNet.

#### 2.5.1 Optimiser

We used an implementation (Kochurov et al., 2020) of the RiemannianAdam (Becigneul & Ganea, 2018) optimiser for all training loops involving SPDNet layers. Aside from learning rate and weight decay, all optimiser parameters were left at default values. Learning rate and weight decay were found through coarse grid searches over typical values; seeSection 2.10for more details.

#### 2.5.2 Network width and channel specificity

Neural network architecture is typically characterised by depth (number of layers) and width (neurons per layer). For the proposed SPDNet based models described in this study, FBSPDNet and EE(G)-SPDNet, depth will be defined by the number of BiMap-ReEig pairs in the SPDNet section,$N_{BiRe}$(seeSection 2.2), and has a static value of 3 for most of the results seen here. This section describes how network width is parametrised in this study.

We define the network width,$N_{f}$, as how many times each input EEG signal gets duplicated during the filtering stage. So for$N_{f}>1$, an individual signal from a single electrode will be filtered multiple times, creating$N_{f}$filtered signals per electrode. Consequently, the number of columns/rows in the covariance matrix size becomes$N_{f}\times N_{e}$, which then defines the size of the first BiMap layer and subsequent layers (since the BiMap compression factor is fixed). Through the addition of additional weights per layer, increasing$N_{f}$potentially enhances each layer’s feature-learning capacity. The models in this study were explored with values of 1 to 8 for$N_{f}$.

In addition to this, channel specificity was explored at the filtering stage. In previous works with convolution networks, such asSchirrmeister et al. (2017), and also in typical filterbanks pipelines such asAng et al. (2012), each filter is applied to*all*EEG signals. In this sense, the filters are*independent*of the EEG signals. This study explored channel-specific filtering (ChSpec) filtering, where filters are learned for and applied to specific electrodes, potentially extracting task-relevant information at the single-electrode level. So, in this study, a channel independent filtering (ChInd) model with$N_{f}=2$learns two filters for*all*electrodes (resulting in$N_{f}$learned filters) and a ChSpec model with$N_{f}=2$learns two filters for*each*electrode (resulting in$N_{f}\times N_{e}$learned filters). This also means that switching between ChSpec and ChInd filtering for a given$N_{f}$does not change the size of the resulting covariance matrix (and therefore does not effect the size of the downstream layers).

### 2.6 SPD matrix operations

This section will briefly outline some of the SPD Matrix operations that are common to most models/pipelines.

#### 2.6.1 Vectorisation

In the “Vect” layer, and when using the rSVM, SPD matrices were vectorised. The vectorisation process is as follows: For$S\in S_{n}^{pd}$, the vectorisation operation,Vect(S)stacks the unique elements ofSinto a$\frac{n}{2}\left(n+1\right)$dimensional vector.^†^To preserve equality of norms, a coefficient of$\sqrt{2}$is applied to the off-diagonals (Barachant et al., 2013).

$$Vect(S)=[S_{1,1},\sqrt{2}S_{1,2},S_{2,2},\sqrt{2}S_{1,3},\sqrt{2}S_{2,3},S_{3,3},...,S_{n,n}]$$ (5)

#### 2.6.2 Concatenation

When constructing the SPD matrices from the filterbank parameters (number of filters,$N_{f}>1$) with channel independent filtering, an SPD matrix was constructed for each filter (a pair of cut-off frequencies for a bandpass filter). These SPD matrices were then concatenated into one larger SPD matrix, before being passed to the next stage of the pipeline.

The following describes a concatenation procedure for multiple SPD matrices (of variable size) that results in a matrix that is provably SPD.^‡^Let$S_{n}$and$S_{m}$be SPD matrices of sizenandm, respectively. Let$Conc(S_{n},S_{m})$be the mapping:$S_{n}^{pd}\times S_{m}^{pd}\Rightarrow S_{n+m}^{pd}$such that the output is a block diagonal matrix of the form:

$$Conc(S_{n},S_{m})=\left(\begin{array}{cc} S_{n} & 0_{n\times m}\\ 0_{m\times n} & S_{m} \end{array}\right)$$ (6)

The size of the concatenated matrix is equal to the sum of the sizes of the input matrices. It is trivial to see how this mapping can be consecutively applied to concatenate any number of SPD matrices. This allows for the simple concatenation of multiple SPD matrices, potentially of different size (although this property is not explored here), in a way that retains the SPD property.

#### 2.6.3 Interband covariance

The above described concatenation procedure allows for the combining of SPD matrices to form a single SPD matrix. As previously mentioned, this is used when creating an SPD matrix for each filter of a channel independent filterbank. Therefore, the block diagonal matrix,C, may be composed of SPD matrices$S_{1}$and$S_{2}$(both of sizen) which are independently computed after filtering trialsTthrough filters$F_{1}$and$F_{2}$(i.e.,$S_{1}$is the covariance matrix of$T_{1}$, which isTpassed through$F_{1}$).

$$C=Conc(S_{1},S_{2})=\left(\begin{array}{cc} S_{1} & 0_{n\times n}\\ 0_{n\times n} & S_{2} \end{array}\right)$$ (7)

InC, the off diagonal blocks are all 0, however, if$T_{1}$and$T_{2}$were first stacked along the electrode axis:

$$T^{\prime }=\left(\begin{array}{c} T_{1}\\ T_{2} \end{array}\right)$$ (8)

one can compute a slightly altered covariance matrix$C^{\prime }$:

$$C^{\prime }=Cov\left(T^{\prime }\right)=\left(\begin{array}{cc} S_{1} & IC\\ IC^{⊤} & S_{2} \end{array}\right)$$ (9)

The off diagonal blocks of$C^{\prime }$,IC, contain the covariance between electrodes of different frequency bands, labelled here as the inter frequency band covariance (simply, the interband covariance), and the diagonal blocks contain, as inC, the within-band covariance. Setting the off diagonal blocks to0turns$C^{\prime }$intoC. Both with and without interband covariance models have been explored in this study.

The interband covariance can only be calculated for models with$N_{B}\geq 2$. This means that in figures showing singleband results, there is only one channel independent model.

#### 2.6.4 Regularisation

During certain analyses or visualisation, estimated SPD matrices required regularisation. When this was needed, matrices were regularised using an ReEig layer. Additionally, the combination of the BiMap and ReEig layers will force non-positive-definite (PD) input matrices to be PD.

### 2.7 Analyses

In the following section, the techniques used for analysis will be detailed. The main methods of analysis used are those that produce the frequency gain spectra figures and the layer-by-layer (LBL) performance assessment. All analysis was performed using the train/test split from the validation set (i.e., no final evaluation set).

#### 2.7.1 Chosen frequency spectra

The Sinc layer used in FBSPDNet and Sinc-EE(G)-SPDNet offers easily interpretable filterbank parameters as sets of high- and low-pass filter cutoff frequencies. When displaying this information, such as inFigure 7, we show the frequency coverage on the y-axis. This is a percentage value that indicates how many of the bandpass filters of that group of models contain that particular frequency, so 75% at 30 Hz indicates that 75% of bandpass filters covered 30 Hz, for that model grouping.

#### 2.7.2 Frequency gain spectra

Visualising the learned filterbank of EE(G)-SPDNet is not so simple, as it uses convolutional filters, which do not directly correspond to a particular region in the frequency space. To assess and visualise the frequencies filtered by the convolutional layer, we calculated the frequency gain spectra by comparing pre- and post-filterbank/convolution frequency spectra. The frequency gain, shown in dB, represents amplification (positive values) or attenuation (negative values) of frequencies relative to the original input (Fig. 5).

The average frequency gain spectra seen in the figures here average across participants, seeds, and filters, and the precise order of calculations and operations performed for the gain spectra is detailed in theSupplementary Materials(Section 1.2). In some cases, certain frequency regions were attenuated so much that their frequency gain became negative infinity. Negative infinity values were usually interpolated, but a handful of spectra with over 50% negative infinities were discarded before plotting.

#### 2.7.3 Peak detection for multiband assessment

The learned filters of EE(G)-SPDNet were analysed to count how many would be classed as “multiband” filters. As is explained in more detail inSections 3.5and4.2.1, a multiband filter is a bandpass filter that lets two or more distinct frequency regions pass. Examples of multiband filters can be seen in the Electrode-Frequency relevance plots inFigure 15.

To analyse the occurrence of multiband filters in EEGSPDNet’s learned filterbanks, we employed a peak detection algorithm on the frequency gain spectra. After discarding/interpolating troublesome spectra (as previously explained), the signals were smoothed with a moving average and zeroed around the median. We then used an out-of-the-box peak detection function (seeSupplementary Materialsfor details) with heuristically set height and width parameters to detect peaks while avoiding noise.

#### 2.7.4 Electrode-frequency relevance

Electrode-frequency relevance, shown inFigures 14and15, combines frequency gain spectra and electrode relevance.

The relevance values for these plots are aggregated gradients calculated from the feature map after the covariance pooling layer, and before the first BiMap layer (i.e., the computed covariance matrix). Gradients of each prediction with respect to the associated covariance matrix in the training set were calculated, summed across rows, and multiplied by the associated normalised frequency gain spectra. This process, while computationally intensive, provides valuable insights into the features discussed in this study.

#### 2.7.5 Layer-by-layer performance

In order to provide some insight into architectural performance, the network was analysed in a layer-by-layer (LBL) manner. This involved passing the data through the trained network, and classifying at every suitable stage in the network. The same preprocessing and data splitting was used here, such that the data exposed to the classifier is the same as the data used by the network. This allows for an understanding of the effect of each layer on classification performance as well as an evaluation of network architecture which may lead to possible areas of improvement. Classification was performed with a Euclidean (SVM) and a Riemannian (rSVM) classifier, to give insight into the data in each of these spaces. Furthermore this may lead to some insights into the internal representation of the data, which is explored in some visualisations.

#### 2.7.6 BiMap gain

In order to visualise the usage of spatial information by the trained networks, we aggregated the weights of the first BiMap layer. This is a linear layer which takes the computed SCM as input. Therefore, it is possible to calculate the contribution of each term in the SCM to the output. We label the contribution of the input to the output the gain.

For an SCMCof size$N_{C}$, output matrixMof size$N_{M}$, and BiMap weightsWwe can writeEquation 2as:

$$M=W^{⊤}CW$$ (10)

Therefore, the$i^{th}$and$j^{th}$component of the output$M_{ij}$is:

$$M_{ij}=\sum_{p=1}^{N_{C}}\sum_{q=1}^{N_{C}}W_{ip}^{⊤}C_{pq}W_{qj}$$ (11)

The gain applied to$C_{pq}$for an output$M_{ij}$is then given by:

$$G_{pq}^{ij}=W_{ip}^{⊤}W_{qj}$$ (12)

Therefore, the gain applied to$C_{pq}$across the whole ofMis:

$$G_{pq}=\sum_{i=1}^{N_{C}}\sum_{j=1}^{N_{C}}W_{ip}^{⊤}W_{qj}$$ (13)

which is the summed outer product of the$p^{th}$and$q^{th}$rows inW:

$$G_{pq}=\sum_{i=1}^{N_{C}}\sum_{j=1}^{N_{C}}{\left(W_{p}⊗W_{q}\right)}_{ij}$$ (14)

The symmetric matrixGthen shows the gain applied to each entry in C. To visualise the spatial gain, we summed G along the rows. This means that the “single electrode” values seen in the plots are the summation of the gains applied to that variance of that electrode plus the gain applied to its covariance with every other electrode.

### 2.8 Comparison models

We have implemented 4 other deep networks for EEG to serve as comparisons to our models in final evaluation. All are deep convolutional networks, and as such also learn convolutional filters (i.e., they learn the filterbanks) during training. Deep4Net (Schirrmeister et al., 2017), ShallowFBCSPNet (Schirrmeister et al., 2017), and EEGNetv4 (Lawhern et al., 2018) are all standard convolutional networks with differing architectures. TSMNet (Kobler et al., 2022) differs from the others in that it is an SPDNet based DRN, originally designed for domain adaptation tasks. The key differences between TSMNet and our proposed models are that$N_{BiRe}=1$for TSMNet and it uses a Riemannian batch normalisation layer, as well as a similar convolutional filtering stage to Deep4Net and ShallowFBCSPNet (temporal followed by spatial filtering).

For Deep4Net, we have not implemented cropped decoding, which will likely decrease it’s classification performance. Regarding TSMNet, we have used the SPD momentum batch normalisation layer (Kobler et al., 2022), as it was the most appropriate to our setting. For other technical aspects of these models, we would direct the reader to their associated papers.

### 2.9 Datasets

We used 5 public motor EEG datasets for this study: Schirrmeister2017 (Schirrmeister et al., 2017), BNCI2014_001 (Tangermann et al., 2012), BNCI2014_004 (Leeb et al., 2007), Lee2019_MI (Lee et al., 2019), and Shin2017A (Shin et al., 2017). All of these were downloaded using MOABB (Jayaram & Barachant, 2018). Table 1, Section 1.8 in theSupplementary Materialslists various data specific details. In general, the datasets underwent minimal preprocessing: scaling, clipping, resampling, and a single bandpass filter. For final evaluation we used the default train/test splits of all datasets. During hyperparamter exploration (i.e., the validation phase), we used a sequential 80:20 split of the training set from Schirrmeister2017. The holdout set/final evaluation set of this dataset was kept “unseen”.

As will be further detailed in the following section, since much of the analysis explores how results vary with different hyperparameter combinations, all of the analysis was conducted on this validation split.

### 2.10 Procedure

This section outlines the experimental procedure, including training loops, data splits, and hyperparameter searches. The results are categorised into two phases: a hyperparameter search phase and a final evaluation phase.

The hyperparameter search phase utilised a validation split of the Schirrmeister2017 dataset to determine learning rate, weight decay, and network design parameters. Analysis was applied to models from this phase to examine how the data changed with these parameters. The best model of each proposed design was selected for the final evaluation phase. In this phase, the models were trained (separately) on the 5 datasets. All datasets were downloaded using MOABB (Jayaram & Barachant, 2018), and had minimal preprocessing applied.

For either phase, every training/testing loop was applied in a*subject-independent*manner and with 3 random seeds. This means that for any given dataset, the network would be trained/tested on a single participant 3 times, each time with a different random initialisation. Scores/analyses were then averaged across participants & seeds to give a single accuracy for that model on that dataset. This approach is also used for the filterbank search done in the FBSPDNet pipeline, resulting in 3 filterbanks per participant (one per seed).

FBSPDNet filterbanks were searched using Bayesian optimization with four objective functions based on two metrics (AIRM and LEM) and two classifiers (rMDM and rSVM). The search was limited to 1000 trials or 12 hours of walltime. The resulting filterbanks were then used to filter the data before covariance matrix computation and SPDNet training. Optimisation was performed on the training set only, making cross-validation (CV) accuracies from FB-Opt classification with rMDM or rSVM not directly comparable to SPDNet scores.

Values for the weight decay and learning rate were found via coarse grid search of typical values with a subset of models. The first gridsearch was used to choose a static value for network depth, parametrised by$N_{BiRe}$, and can be seen inSupplementary Figure 13. The search used an 8-filter ChSpec EE(G)-SPDNet, and a value of$N_{BiRe}=3$was brought forward for all other variants of FBSPDNet and EE(G)-SPDNet. A Sinc variant of the same model was used in another search to set the values of learning rate and weight decay for the EE(G)-SPDNet models (Supplementary Fig. 14). Next, weight decay and learning rate values for the 4 FBSPDNet variants were also searched, using a channel-specific 8 Filter model (Supplementary Fig. 15), and these values were kept for any other variants of the FBSPDNet. Finally, the weight decay and learning rates for the 4 comparison models were also searched (Supplementary Fig. 12). These are the learning rate and weight decay values used for the remainder of computations.

The proposed models were then trained/tested for a range of network widths,$N_{f}\in 1,2,3,4,5,6,7,8$(much of the analysis was performed on the data acquired during this stage). It is with these results that the best EE(G)-SPDNet and best FBSPDNet were chosen and brought forwards to final evaluation. They were then trained/tested on the final evaluation datasets using the hyperparameters found previously, and compared against the other out-of-the-box models.

For both phases, classification accuracy refers to unseen test set performance, with the exceptions of the tSNE plots (Figs. 12and13) and the electrode-frequency relevance plots (Figs. 14and15) where both training set and test set accuracies are given.

### 2.11 Statistics

In some results/figures, p-values are given to assess the significance of the model comparisons. For these tests, the Wilcoxon signed-rank test (seeSupplementary Section 1.6for package details) was used.

For the heatmap displayed inFigure 3, accuracies were averaged across seeds. It is from these scores that the mean differences and p-values were calculated.

**Fig. 3.**
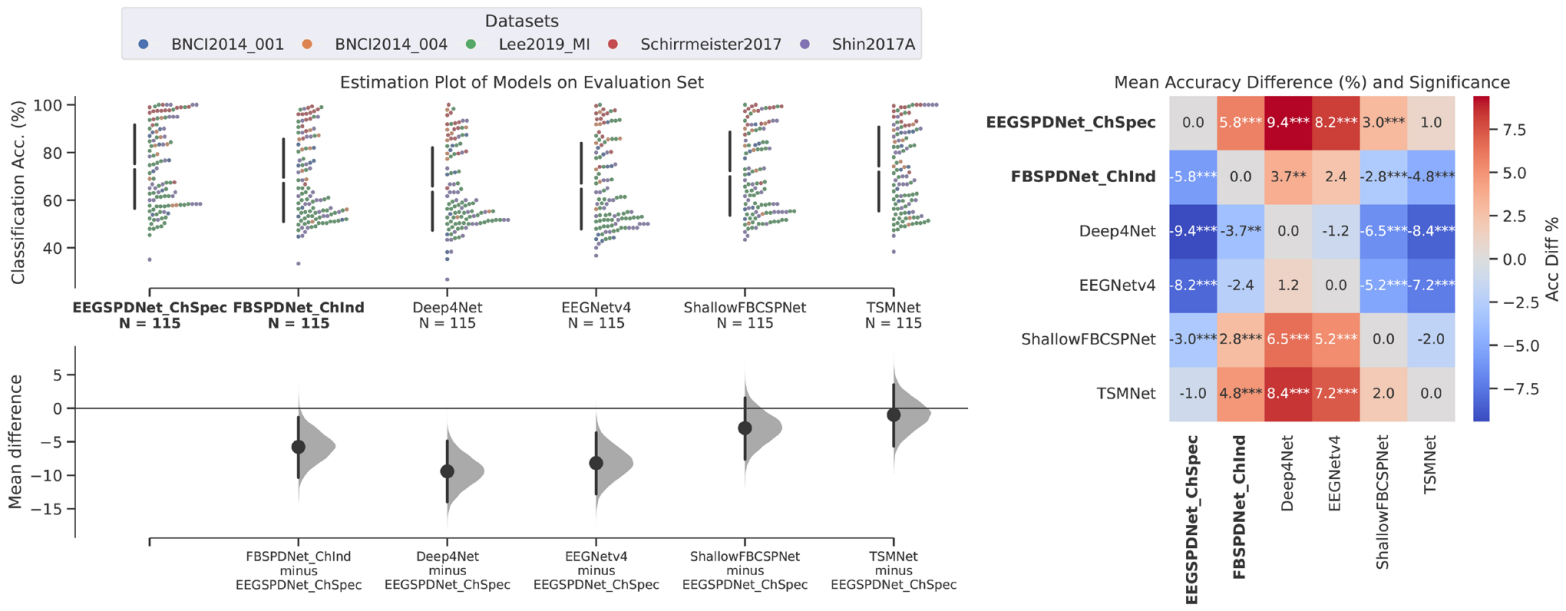
Final evaluation set results. These figures show the results of our models (EEGSPDNet and FBSPDNet) and the comparison models on the evaluation datasets. Details regarding the comparison models we used can be found inSection 2.8, and details regarding the datasets can be found inSection 2.9. The variants of our proposed models are an 8-filter, channel-specific Conv-EE(G)-SPDNet (EEGSPDNet ChSpec) and a 6-filter channel independent FBSPDNet (FBSPDNet ChInd). On the left is an estimation plot, which is itself made up of two subplots. The upper subplot is a swarm plot, with the models on x-axis, and the test-set classification accuracy on the y-axis. Each point represents a single participant training-testing loop from a single dataset (hue denotes which dataset) for a given model. To the left of a particular models swarm is a gapped line showing the swarm mean +/- standard deviation. There are 115 points in each swarm, which is the number of participants across all datasets. The lower subplot shows the bootstrapped (n=10000) mean difference between the left-most model (EEGSPDNet ChSpec) and every other model. The shaded area shows the distribution of the bootstrapped differences, with the dot and line respectively showing the mean and 95% confidence intervals. On the right is a heatmap displaying mean accuracy differences (not bootstrapped, as in the estimation plots) and asterisks for significance thresholds (one, two, or three asterisks imply significance less than 0.05, 0.01, & 0.001, respectively). A cell in the heatmap represents the row minus column accuracy difference.

ForTables 3,4and5, which respectively pair with the left, centre and right subplots ofFigure 4, values were averaged across seeds and participants to give a single accuracy per value of$N_{f}$. It is from these scores that means, standard deviations, and p-values were calculated.

**Table 3.** FBSPDNet versus EE(G)-SPDNet averaged across.

Filter specificity	EE(G)-SPDNet (Conv)	FBSPDNet	p-Value
Channel Independent	89.8 +/- 5.2	92.4 +/- 3.4	0.0078125
Channel Specific	93.9 +/- 3.0	91.8 +/- 2.6	0.0078125

$N_{f}$This table shows mean +/- std values for the data displayed inFigure 4(left).

**Table 4.** ChInd versus ChSpec averaged across.

Model	Channel independent	Channel specific	p-Value
Conv-EE(G)-SPDNet	88.3 +/- 5.4	94.6 +/- 2.2	0.0078125
Sinc-EE(G)-SPDNet	91.3 +/- 5.1	93.2 +/- 3.8	0.0078125
FBSPDNet	92.4 +/- 3.4	91.8 +/- 2.4	0.1953125

$N_{f}$This table shows mean +/- std values for the data displayed inFigure 4(centre).

**Table 5.** With versus without interband covariance averaged across.

EEGSPDNet filter type	With interband	Without interband (RmInt)	p-Value
Convolution	88.3 +/- 5.4	86.4 +/- 4.5	0.015625
Sinc	91.3 +/- 5.1	90.1 +/- 5.1	0.015625

$N_{f}$This table shows mean +/- std values for the data displayed inFigure 4(right).

**Fig. 4.**
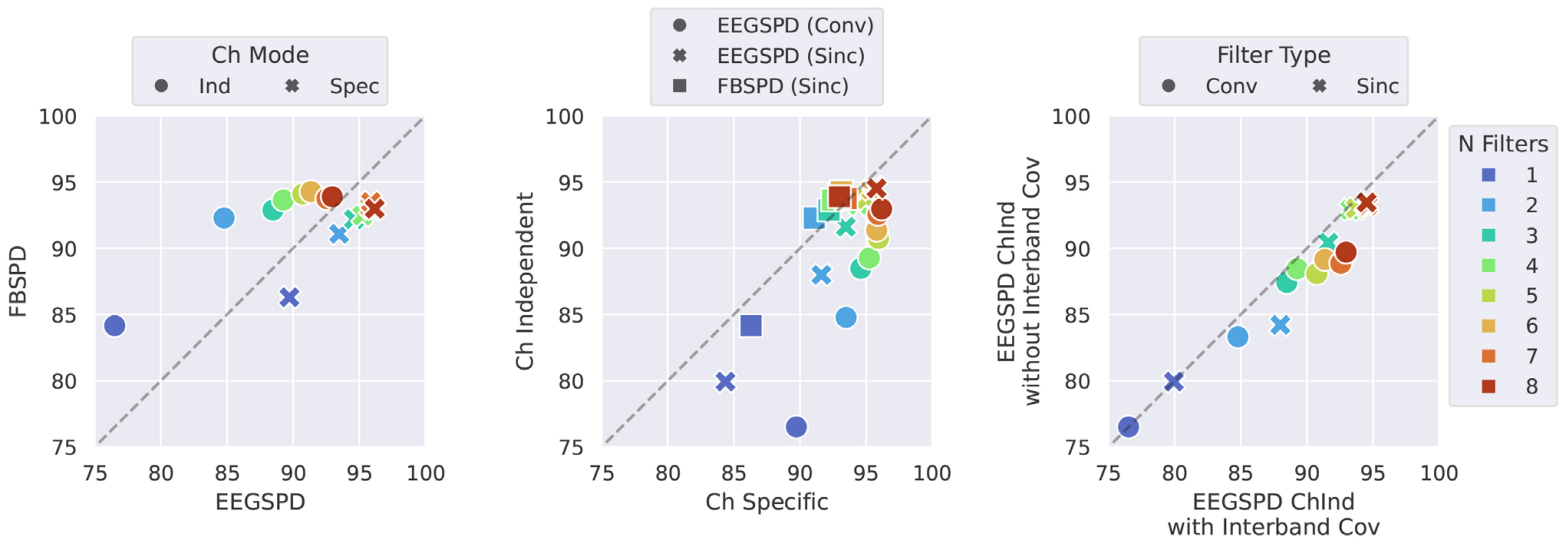
Validation set classification accuracy comparison for different conditions. From left to right, the subplots show: FBSPDNet against EE(G)-SPDNet with marker denoting ChInd or ChSpec filtering, ChInd against ChSpec filtering with marker denoting EE(G)-SPDNet or FBSPDNet and removed interband covariance against with interband covariance with markers denoting regular conv filtering or sinc-conv filtering. Data have been averaged across seeds and participants, and separated by number of filters (via colour). Means, standard deviations and p-values for the three subplots can be found inTables 3,4, and5. The similarity between many p-values arises from the relatively small sample size (7 or 8 values) and that the ranked sums are often identical (i.e., all points are in favour of a particular condition).

In other results, we display model comparisons using estimation plots, using the python package ‘dabest’ (Ho et al., 2019).

## 3 Results

In the following section, we detail our experimental results in 8 findings. The first 3 of which are focussed on the classification performance between models. The remaining 5 findings concern patterns in network behaviour that we observed through our analyses. Lastly, we present some visualisations of some specific data, which demonstrates interesting trends, but does not necessarily possess the same robustness as the previous findings.

### 3.1 Finding 1: Deep Riemannian Networks with learnable filterbanks can outperform ConvNets

Figure 3andTable 2illustrate the evaluation set performance of the best performing EE(G)-SPDNet and FBSPDNet models across 5 datasets (seeSection 2.9), and compared with 4 other models (seeSection 2.8). The variants of our proposed models brought forwards to the evaluation stage are an 8-filter, channel-specific Conv-EE(G)-SPDNet (EEGSPDNet ChSpec) and a 6-filter channel independent FBSPDNet (FBSPDNet ChInd). EEGSPDNet demonstrated superior performance, achieving statistically significant improvements in classification accuracy against the FBSPDNet model, Deep4Net, ShallowFBCSPNet, and EEGNet, with a small non-significant increase against TSMNet. In contrast, FBSPDNet showed more modest improvements, outperforming only Deep4Net and EEGNet, with only the former at some statistically significant level. Overall, the DRNs (EEGSPDNet, FBSPDNet, and TSMNet) generally outperformed the non-Riemannian models. Additionally, ShallowFBCSPNet was the best-performing non-Riemannian model, achieving a statistically significant accuracy increase against FBSPDNet, Deep4, and EEGNet. Lastly, TSMNet’s performance was marginally below EEGSPDNet’s, with slightly less statistical significance against the other models.

### 3.2 Finding 2: Highest performance achieved by a wide, convolutional, channel-specific, DRN

The best-performing model overall was an 8 filter, channel-specific EE(G)-SPDNet using a standard convolutional filter. It achieved the highest scores on the validation set (seeFig. 4(left)), and was therefore brought forward to the evaluation set phase on 5 datasets, where it also achieved the highest accuracies (seeFig. 3). The highest-performing FBSPDNet model on the validation set (seeFig. 4(left)) was a 6 filter channel independent model, although it failed to achieve notable performance on the evaluation datasets. While these two models had differing preferences for channel specificity, both showed that generally wider networks (i.e., more filters) perform better (seeFig. 4(left and centre)). However, this effect appears to plateau for FBSPDNet at$N_{f}=6$(seeFig. 4(left)).

As previously stated, the best-performing model was the 8-filter Conv-EE(G)-SPDNet with channel specific filtering. In fact, the Conv-EE(G)-SPDNet outperformed the Sinc-EE(G)-SPDNet and the 4 FBSPDNet variants across all$N_{f}$in the ChSpec setting (Supplementary Fig. 2). However, this trend was reversed in the ChInd setting (Supplementary Fig. 1), where the Conv-EE(G)-SPDNet was outperformed by the Sinc-EE(G)-SPDNet, and both were beaten by some variants of the FBSPDNet (the ones that used the rSVM as their proxy classifier for the FB-Opt). These results suggest that both ChSpec filtering and convolutional filters were crucial for achieving the highest decoding accuracies.

### 3.3 Finding 3: Including covariance between frequency bands can boost accuracy

As can be seen inFigure 4(right), the removal of the interband covariance negatively affected performance for both Conv- and Sinc- EE(G)-SPDNet. This effect appeared the strongest in the Conv-EE(G)-SPDNet.

### 3.4 Finding 4: Learned frequency profile is physiologically plausible

**Fig. 5.**
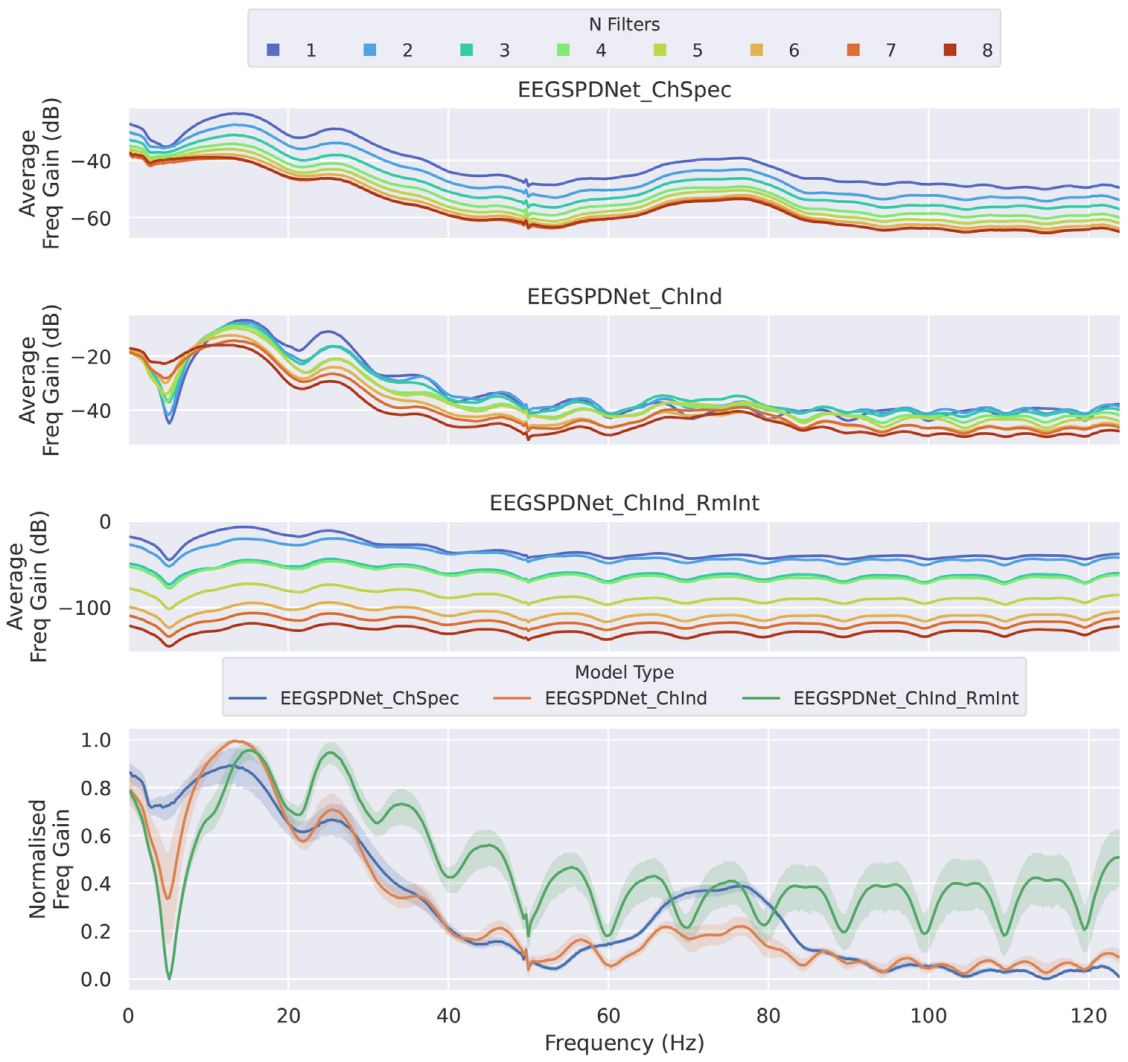
Average frequency gain caused by learned convolutional filters of Conv-EE(G)-SPDNet. Each subplot shows the average frequency gain (in decibels) across the spectrum. The first three subplots (from top to bottom) show the different model sub-types, with colour denoting the number of filters. The bottom subplot shows the average (with standard deviation in shading) across all filters for each model sub-type; frequency gains were normalised between 0–1 to allow for better inter-model comparisons. All data used for the generation of this figure were collected during the validation phase.

The frequencies selected by the optimised filterbanks were also analysed. Frequency gains resulting from the convolutional filter training (Fig. 5) and the frequency regions selected by the optimiser in the FB-Opt stage of the FBSPDNet (Fig. 7; Supplementary Section 1.5) exhibited prominent peaks in three distinct ranges: 8–20, 20–35, and 65–90 Hz. These ranges correspond to the alpha, beta, and high-gamma bands, respectively. Notably, classifiers incorporating features from the high-gamma band demonstrated superior performance overall. These areas are consistent with the areas activated by motor movement (Ball et al., 2008;Pfurtscheller & Neuper, 2001). Additionally, manual inspection of particular electrodes in the electrode-frequency relevance plots (Figs. 14and15) suggests that the networks also select physiologically plausible spatial regions.

**Fig. 6.**
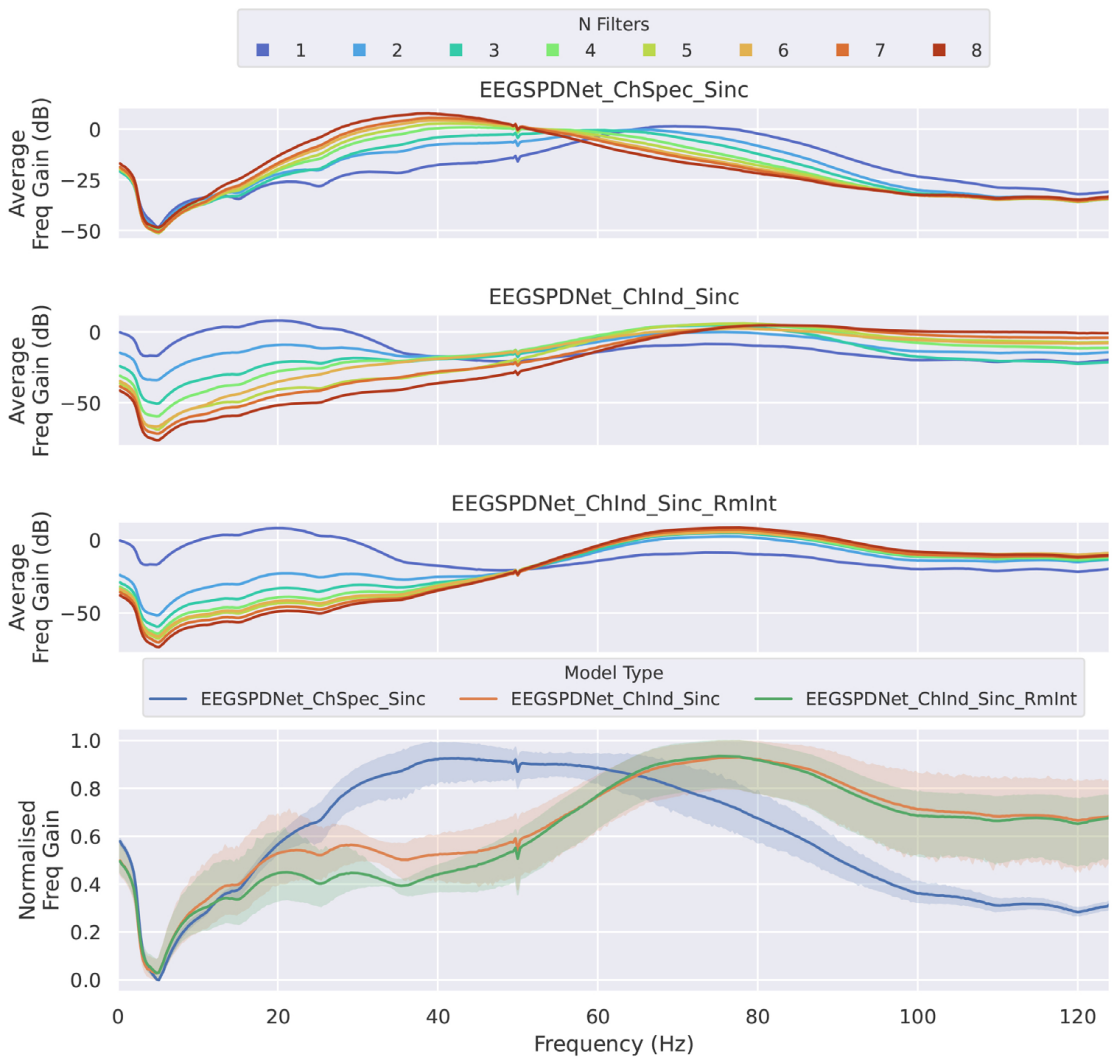
Average frequency gain caused by learned convolutional filters of Sinc-EE(G)-SPDNet. Each subplot shows the average frequency gain (in decibels) across the spectrum. The first three subplots (from top to bottom) show the different model sub-types, with colour denoting the number of filters. The bottom subplot shows the average (with standard deviation in shading) across all filters for each model sub-type; frequency gains were normalised between 0–1 to allow for better inter-model comparisons. All data used for the generation of this figure were collected during the validation phase.

**Fig. 7.**
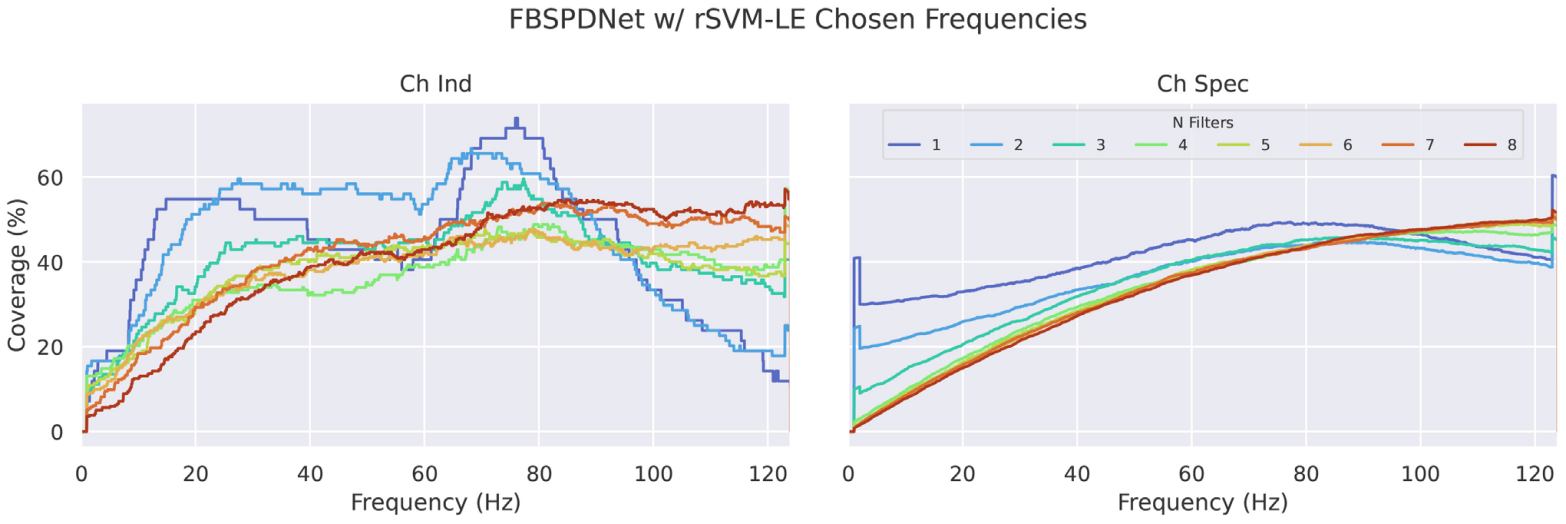
Frequency band selection distributions for FBSPDNet. Each subplot shows the selected frequency distribution across the spectrum, left for ChInd and right for ChSpec. The colour of the line denotes the number of filters for that model. A value of 100% means that frequency was included in every bandpass filter (including every participant and if channel specific, and every electrode); seeSection 2.7.1for precise details. All data used for the generation of this figure were collected during the validation phase.

### 3.5 Finding 5: Conv-EEGSPDNet models may learn task-relevant multiband filters

**Fig. 8.**
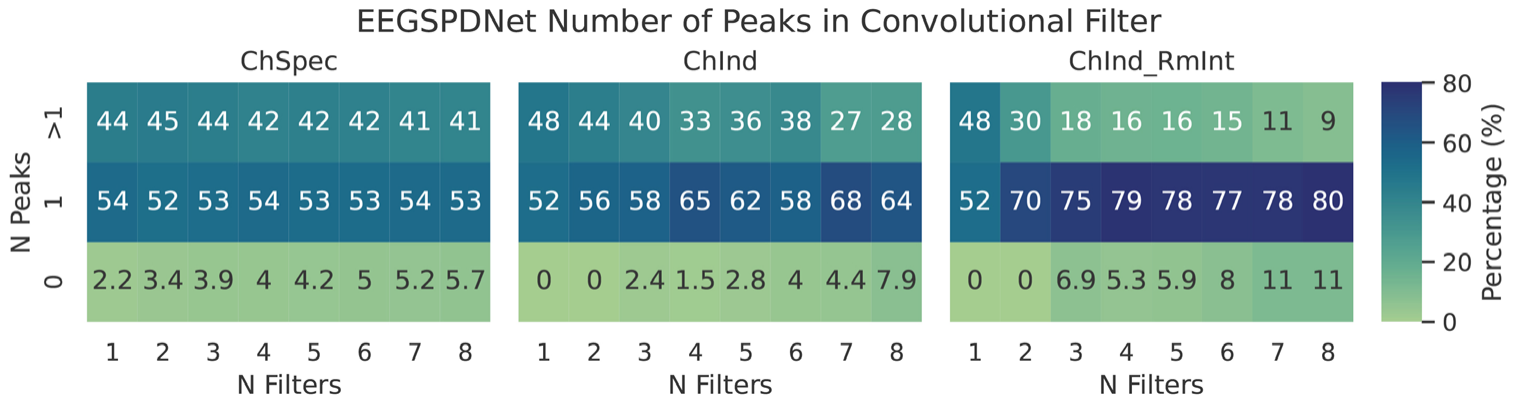
EEGSPDNet multiband occurrence via number of peaks in frequency gain. For every convolutional filter, the numbers of peaks in the frequency gain spectra were counted. Exact details of peak detection can be found inSection 2.7.3. Each subplot represents a different model sub-type, ChSpec (left), ChInd (center), and ChInd with interband covariance removed (right). The x-axis shows the number of filters,$N_{f}$, for the given model. The y-axis shows the category of number of peaks found in the response of each filter (0, 1, or>1 peaks). The colour/annotation of each cell shows the percentage of frequency gain spectra that fell into number of peaks category for the given model (subplot) and$N_{f}$(x axis). Therefore, any column in a heatmap sums to 100, and a value of 70 for$N_{f}=2$and N Peaks = 1 indicates that 70% of that models frequency gain spectra had exactly 1 peak. Data used was all from the validation phase.

The numbers of peaks present in the frequency gain spectra were also analysed, suggesting that the networks learn “multiband” filters. These are filters that preserve/amplify more than 1 distinct region in the frequency space.Figure 8shows that all variants of the Conv-EE(G)-SPDNet learned multiband filters, with the ChSpec variant having the largest proportion of multiband filters. The electrode-frequency relevance plots provided some examples of specific multiband filters, which can be seen most prominently inFigure 14(right hand, FFC6h) where the model has learned a filter that selected alpha/beta and high gamma specifically.

### 3.6 Finding 6: Only initial ReEig layers were active

The eigenvalues of the feature map matrices inside the some EE(G)-SPDNet model were also computed. This data are presented inFigure 9and show that the majority of eigenvalues in the feature map matrices are above the ReEig activation threshold before reaching the first ReEig layer, and none remain after this first eigenvalue rectification. In this sense, the first BiMap-ReEig pairing rectifies all eigenvalues for the rest of the network. It can also be observed that network width increased the presence of below-threshold eigenvalues. Despite this, the initial parameter sweeps (Supplementary Fig. 13) still indicated that a network with 3 pairs of BiMap-ReEig was ideal.

**Fig. 9.**
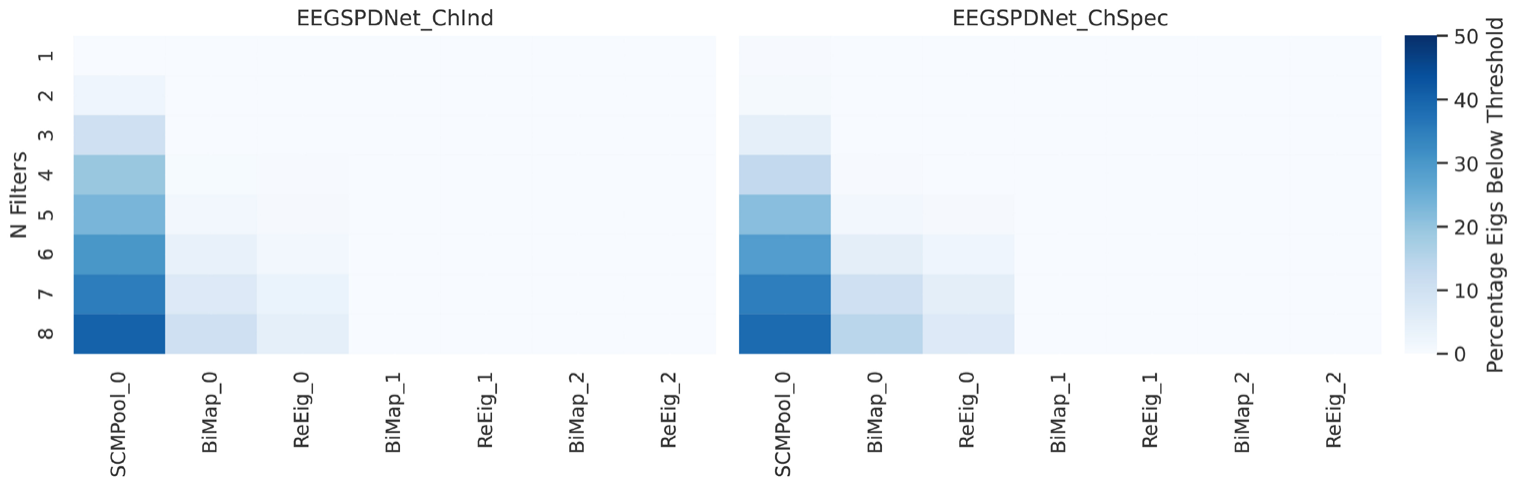
Percentage of eigenvalues below ReEig threshold. Heatmaps showing proportion of eigenvalues in feature maps that are below the ReEig threshold (and would, therefore, be rectified).$N_{f}$is shown on the y-axis, and layer name is given on the x-axis. Eigenvalues were calculated after passing the data through the labelled layer.

### 3.7 Finding 7: For filterbank optimisation, classifier choice was more important than Riemannian metric

The estimation plots inFigure 10compare the effect of parameter choices (metric and classifier) at the FB optimisation stage on both the final SPDNet performance and the CV performance of the classifier used for optimisation. The datasets used during CV and for the SPDNet are different (seeSection 2.10) and so direct comparisons between the rSVM/rMDM and the SPDNet are not warranted. From these data, we can see that the choice of Riemannian metric (LE or AIRM) was essentially irrelevant for final SPDNet classifier performance, even though the AIRM produced marginally better results during the FB-Opt stage However, the choice of classifier (rMDM or rSVM) had a much larger impact. The rSVM produced much higher accuracies at the FB-Opt stage and while the magnitude of this effect was not replicated with the ensuing SPDNet, there was still a large difference between the two.

**Fig. 10.**
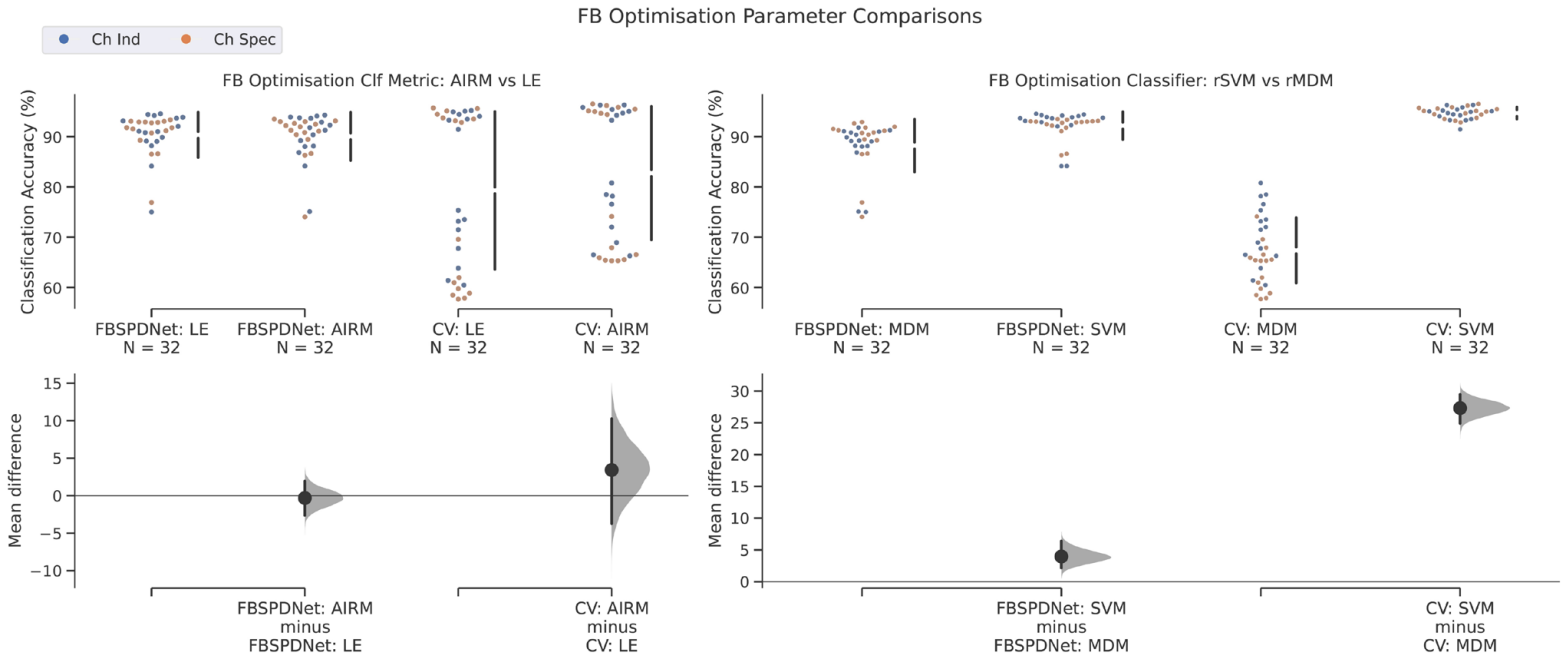
Filterbank optimisation estimation plots. Gardner-Altman Estimation plots showing classification results for the CV stage during filterbank optimisation, and also for final FBSPDNet classification. Both classifiers (rMDM and rSVM) are shown, as well as both Riemannian metrics (LEM and AIRM). For details regarding estimation plots seeFigure 3, and alsoHo et al. (2019).

### 3.8 Finding 8: Networks did not utilise all internal task-relevant information

The layer-by-layer classification analysis (seeFig. 11) revealed that the networks in general failed to sufficiently utilise all task-relevant information. At least one layer (sometimes all), and every model had its final layer classification performance beaten by an rSVM applied on that particular layer’s feature maps of matrices. Generally, these accuracy differences are less than 1%, although some (particularly at lower network width) are more stark. Additionally, there is contrasting behaviour regarding the performance of the regular (euclidean) SVM in this analysis. The SVM never outperformed the final layer SPDNet classification, but the patterns of underperformance do vary between model types. Looking at the EE(G)-SPDNet ChSpec (11), the performance of the SVM was particularly bad in the final layers of the network. This trend is more prominent for wider networks. However, this trend is in contrast to the other network types (seeSupplementary Materials;Figs. 5,6,7, and8), where SVM performance is either fairly constant or improves throughout the network. Furthermore, when looking at$N_{f}=1$, the SVM performed noticeably worse on the feature maps of the EE(G)-SPDNet ChSpec, something which is much less prominent for the other EE(G)-SPDNet models.

**Fig. 11.**
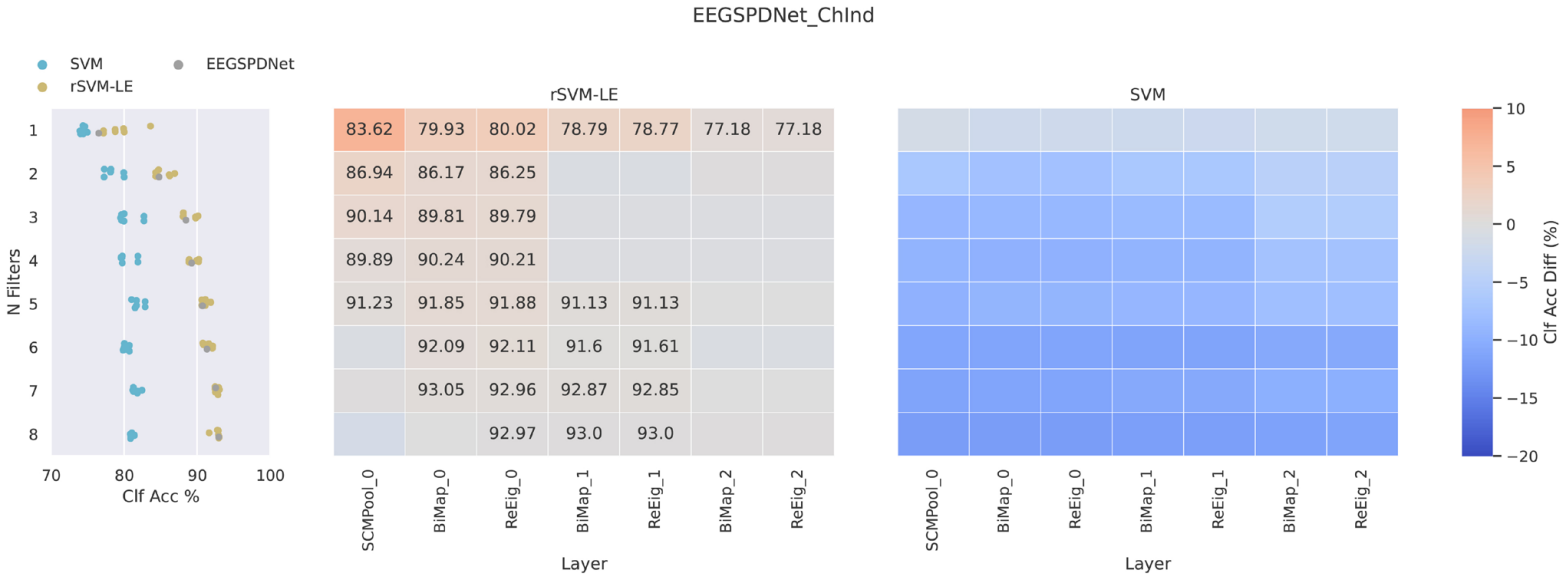
LBL performance of the channel-specific EE(G)-SPDNet. The left subplot column contains swarm plots showing the final accuracy of the associated EE(G)-SPDNet (grey dots) and the absolute performance of the rSVM (blue dots) and SVM (yellow dots), separated by number of filters. The heatmaps (centre and right subplots) show the accuracy difference of the network to an rSVM or regular (euclidean) SVM, respectively. The red/blue shading of each cell shows accuracy difference between the rSVM/SVM and the EE(G)-SPDNet (rSVM/SVM score minus EE(G)-SPDNet score). In the cases where the rSVM or SVM outperformed its associated EE(G)-SPDNet, its absolute accuracy is annotated in the cell. Analysis was performed using data from the validation phase.

An additional LBL calculation on TSMNet did not show the same effect (seeSupplementary Materials;Fig. 9).

### 3.9 Visualisations

#### 3.9.1 tsne

**Fig. 12.**
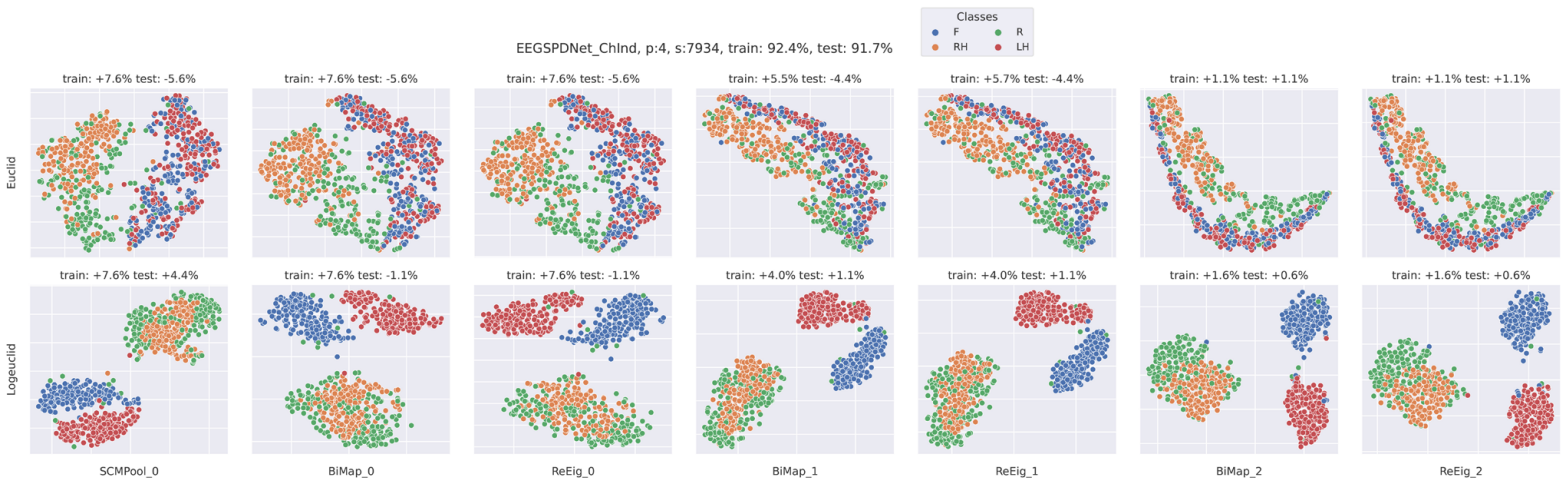
tSNE visualisation of data in channel independent EE(G)-SPDNet. Data is visualised as it passes through the layers of a ChSpec EE(G)-SPDNet. Each column shows the data after it has passed through the associated layer (subplot title). The top row used the Euclidean distance between matrices as the metric, and the bottom used a Riemannian metric (LEM). The final training and testing accuracies of the data are given in the Figure title. The data plotted are test-set data (although from the train/test split of the validation set). The accuracy shown in each subplot title is the relative accuracy of an SVM applied to the data in the subplot. A positive accuracy implies that the SVM outperformed the final EE(G)-SPDNet accuracy. For the top row, a Euclidean SVM was used, and the bottom row used a Riemannian SVM (Log-Euclidean). The colour of each point in the subplots denotes its class label (right hand, left hand, rest or feet) which is also shown in the figure legend. The data shown are those from a single participant for a single model, namely participant 4 from an$N_{f}=1$channel independent model.

**Fig. 13.**
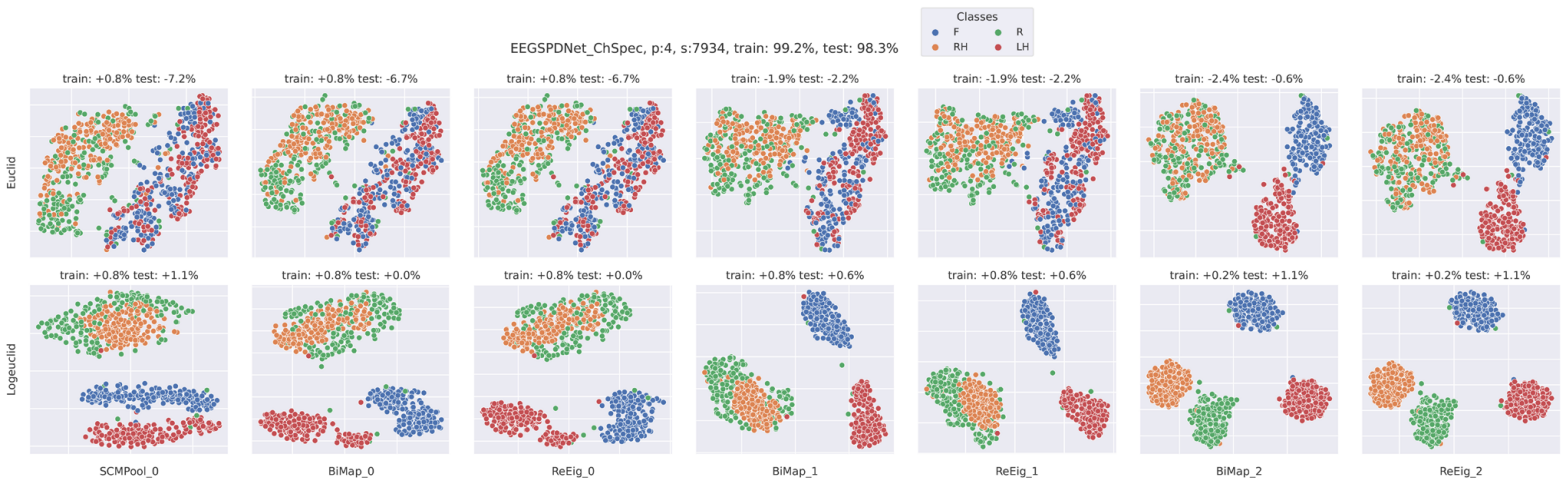
tSNE visualisation of data in channel-specific EE(G)-SPDNet. SeeFigure 12for more details. The data shown are those from a single participant for a single model, namely participant 4 from an$N_{f}=1$channel-specific model.

We also visualised the LBL data using tSNE; two examples of this are shown inFigures 12and13, and both are$N_{f}=1$EE(G)-SPDNet models. We have chosen a high-performing participant and seed, and shown the ChSpec (Fig. 13) and ChInd (Fig. 12) variants.

First, we can see that the participant-seed data shown inFigure 13are somewhat representative of the average accuracy trends shown in the LBL analysis inFigure 11. The rSVM classification accuracies (lower row ofFig. 13) are at a minimum equal to the final SPDNet score, and many of the layers outperform the final layer accuracies. In this particular example, the highest outperformance occurs at the first layer and the last two, instead of just the first layer as in the LBL. The SVM scores stay below final layer classification, but do improve through the network especially after the second BiMap layer. This does not exactly match the overall trend seen in the LBL where Euclidean accuracies stay roughly constant.

In the ChInd setting, the tSNE data inFigure 12with the rSVM match the trend seen in the LBL data in Figure 6 of theSupplementary Materials. The rSVM outperforms the EE(G)-SPDNet strongly early in the network with the effect decreasing as the data are passed through the layers. This is partially observable in the tSNE data itself, as the visual separation between classes barely changes through each layer. The SVM scores inFigure 12are slightly atypical in that the final two layers have outperformed the final layer of the SPDNet. The LBL data suggest that, in general, the SVM does not perform better than the final layer of the SPDNet, and that this does not change much as the data are propagated through the network.

When comparing the two tSNE Figures, we are comparing the ChInd (Fig. 12) filtering to ChSpec (Fig. 13), as all other variables (participant, seed etc) are held constant. It is difficult to draw solid conclusions from the tSNE visualisations, however it is interesting to note how the visual separation of classes varies between these two Figures. The ChSpec model succeeds in separating classes that are not visually separated in the ChInd variant, but only in the final two layers. By the end of the ChSpec network, all four classes are visually separated for the Riemannian tSNE, whereas “Rest” and “Right Hand” are still mixed for the ChInd at the same point in the network. A similar pattern occurs for the Euclidean tSNE, except with “Feet” and “Left Hand”.

#### 3.9.2 Electrode-frequency relevance

**Fig. 14.**
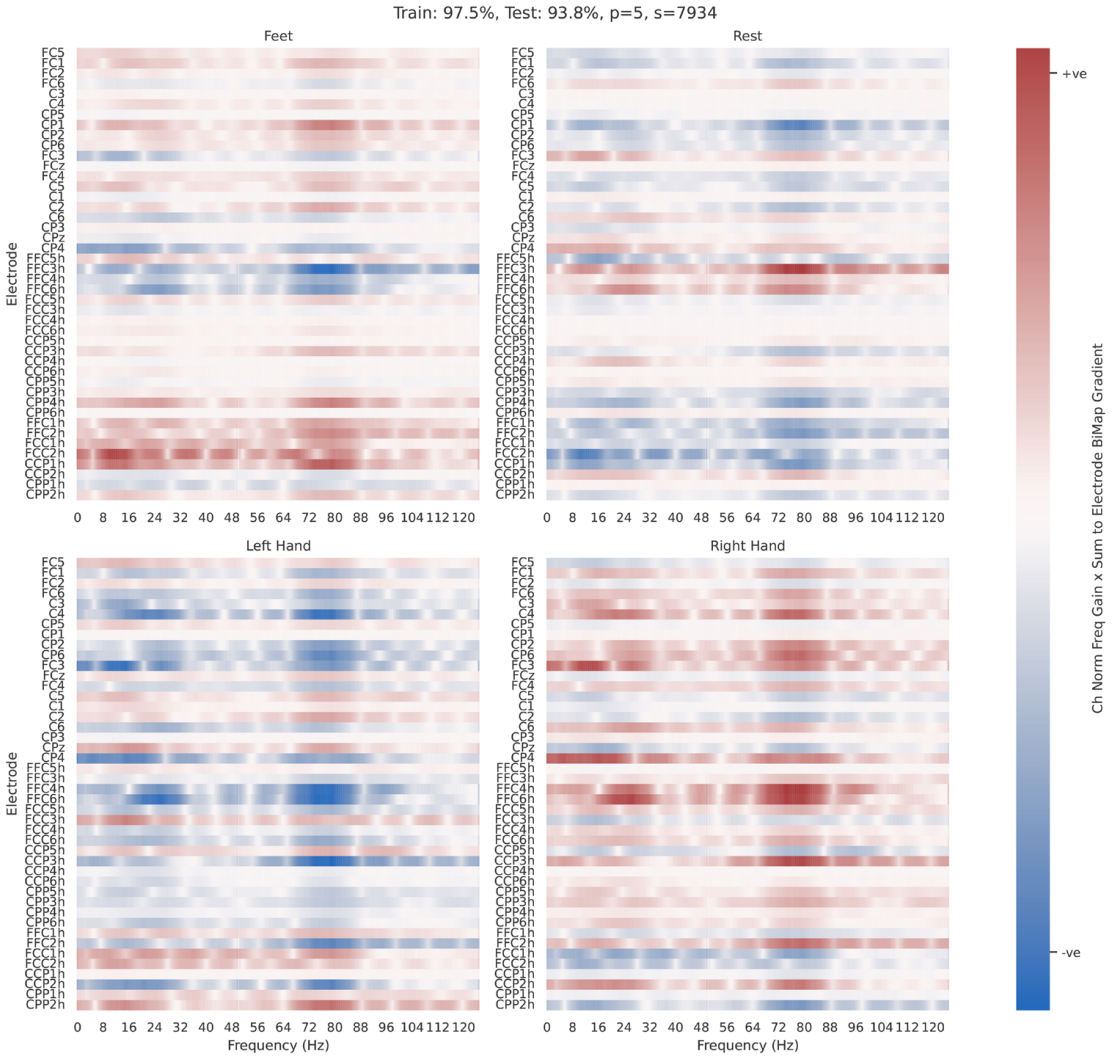
Electrode frequency relevance plots for participant 5, Schirrmeister2017. Per class heatmaps that show the product of normalised frequency gain spectra with aggregated-to-electrode gradient values. Precise details regarding calculations can be found inSection 2.7.4. Each subplot shows each class, with frequency on the x-axis, and electrode number on the y-axis. Hue indicates relevance, that is, a change in that frequency, at that electrode results in a positive or negative increase in the softmax prediction for that class. Data are shown for a single, fully-trained EE(G)-SPDNet model (one participant, one seed), details of which can be seen in the figure title. The model used ChSpec filtering and had$N_{f}$

Figures 14and15show two electrode frequency relevance plots for ChSpec EE(G)-SPDNet,$N_{f}=1$. These two runs were chosen as they highlight a number of interesting features. First, a number of patterns emerge which are typical for subject-specific motor EEG data such as inverted left and right side usage and subject-specific spatial and frequency usage. In particular, we note the usage of the high-gamma region, which is dominant inFigure 14, and absent inFigure 15. Second,Figure 14also shows a strong example of the multiband filter (Electrode FFC6h) selecting two distinct and physiologically plausible frequency region.

**Fig. 15.**
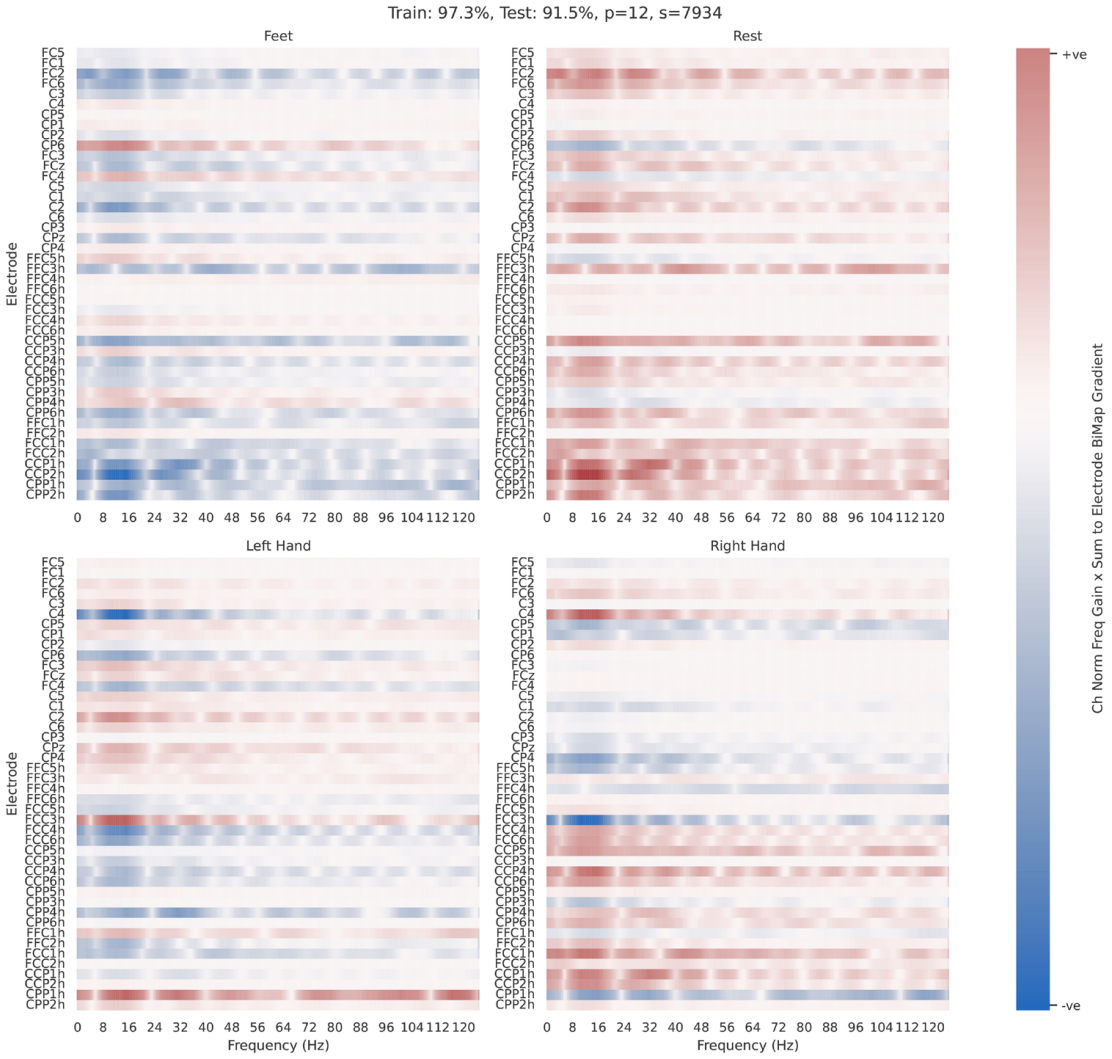
Electrode frequency relevance plots for participant 12, Schirrmeister2017. SeeFigure 14for plot details. The model used ChSpec filtering and had$N_{f}=1$

#### 3.9.3 BiMap gain

InFigure 16, we visualise the gain applied to covariance matrix elements as topographical head plots. These plots highlight the similarity in spatial gain between models with and without interband covariance, and also the spatial dissimilarity of the ChSpec model.Supplementary Figure 3(left) shows the correlation between these topographical headplots, quantifying the above statements.

**Fig. 16.**
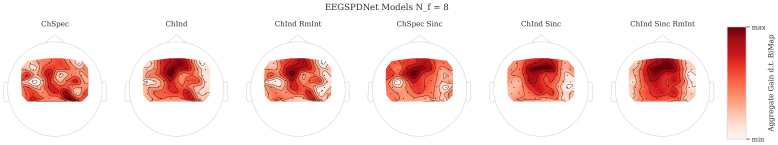
BiMap gain for$N_{f}=8$EEGSPD models. Head plots showing aggregate electrode covariance gain from the first BiMap layer of trained models. For an input covariance matrix, an equally sized gain matrix can be computed which shows the contribution of each element in the covariance matrix to the output matrix. This symmetric can be row (or column) summed to get the contribution of an individual electrode (via its variance and covariance pairs). SeeSection 2.7.6for more details. The colourbar min-max values were set per plot, meaning that absolute comparisons between models are not possible.

## 4 Discussion

### 4.1 Deep Riemannian Networks (DRNs) versus ConvNets

The results presented inFigure 3andSection 3.1strongly suggest that DRNs can outperform the convolutional neural networks on motor-EEG datasets. The only ConvNet model to outperform a DRN was ShallowFBCSPNet, and it was still beaten by EE(G)-SPDNet and TSMNet. In particular, the 8-filter channel-specific Conv-EE(G)-SPDNet has the highest accuracy and most significant results, and from a pure decoding ability perspective, and could be seen as the “best” of the models presented in this study.

### 4.2 Hyperparameter and network design choices and their influence on behaviour

#### 4.2.1 Filter type

This study employed two types of learned filterbanks: FBSPDNet and Sinc-EE(G)-SPDNet used learnable sinc convolutions, while Conv-EE(G)-SPDNet utilised standard convolutional filters. The latter can develop into more complex forms, such as dual-bandpass or multibandpass filters, allowing multiple frequency bands to pass through. Our analysis of multibandpass filter occurrence aimed to investigate performance disparities between Conv-EE(G)-SPDNet and Sinc-EE(G)-SPDNet, which, aside from filtering style, are identical. While other complex filter types may exist, they were not explored in this study.

Manual examination of frequency gain spectra (e.g.,Fig. 14, electrode FFC6h), and a peak detection algorithm (Section 2.7.3) revealed the presence of multiband filters. Results are presented inFigure 8and Finding 5 (Section 3.5). Note that the peak detection algorithm’s parameters were developed heuristically, potentially affecting the accuracy of multiband filter counts.

Several correlations between multiband filters and decoding performance can be observed in the data. Conv-EE(G)-SPDNet’s superior performance over Sinc-EE(G)-SPDNet suggests an advantage in exploring beyond sinc-space filters. Channel-specific Conv-EE(G)-SPDNet models exhibited more multibands and better performance than ChInd models. The best-performing model had a high proportion and absolute number of multiband filters. Interestingly, while the proportion of multiband filters decreased with network width, their absolute number increased, correlating with improved decoding performance.

However, in the ChInd setting, Sinc-EE(G)-SPDNet outperformed Conv-EE(G)-SPDNet, suggesting that the multiband filters present in Conv-EE(G)-SPDNet may have degraded decoding performance, in this context.

In conclusion, multiband filters were identified in Conv-EE(G)-SPDNet’s learned filterbanks, but their precise impact on decoding performance remains unclear beyond the observed correlations. Further investigation is warranted, with potential methodologies outlined inSection 4.5.

#### 4.2.2 Channel specificity

The study’s best classification performance came from an 8 filter ChSpec setup paired with standard convolutional filters, outperforming ChSpec sinc/bandpass filters (Findings 1 & 2). Channel-specific configurations increase network complexity without increasing width, allowing per-electrode frequency region selection, especially at higher network widths. However, not every model benefited from ChSpec filtering, namely FBSPDNet performed better with ChInd filtering at$N_{f}\geq 2$(Fig. 4(center)). In ChSpec settings, Conv-EE(G)-SPDNet outperformed Sinc-EE(G)-SPDNet, while the opposite was true for ChInd settings (Supplementary Figs. 1, 2 and 3(right)).

The reasons for superior performance requiring both channel specificity and convolutional filtering remain unclear. The complex filter types available Conv-EE(G)-SPDNet may be the most effective at the per-electrode level, which is potentially supported by the data at$N_{f}=1$(Fig. 4(center)), where the largest improvement due to ChSpec filtering can be observed. This suggests only a subset of electrodes may require complex filters for optimal decoding. The increased number of multiband filters in ChSpec models could support this theory, though it likely also correlates with the overall filter count. While ChSpec Conv-EE(G)-SPDNet performed best overall, Sinc-EE(G)-SPDNet and FBSPDNet outperformed it in ChInd settings. Additionally, no ChInd EE(G)-SPDNet model was brought forwards to the evaluation set stage, so it becomes difficult to ascertain the exact role of channel specificity in Conv based DRNs for multiple datasets.

TSMNet, which uses channel independent filtering (seeTable 1), achieved comparable accuracies to ChSpec EE(G)-SPDNet. However, TSMNet’s additional features (spatial filtering and SPD batch normalisation) make it difficult to isolate the impact of specific layers on decoding accuracy.

Channel independent models with deleted interband covariance showed slightly lower performance, indicating the positive role of interband covariance in classification. Channel-specific filtering naturally includes interband covariance for models with$N_{f}>1$.

#### 4.2.3 Filterbank optimisation for FBSPDNet

Despite FBSPDNet’s modest decoding accuracies, the filterbank optimisation analysis yielded noteworthy insights.Figure 10and Finding 7 (Section 3.7) show that the choice of Riemannian metric (LEM vs. AIRM) minimally impacted FBSPDNet decoding and only slightly affected rSVM decoding (AIRM performed marginally better). This suggests that the Riemannian metric choice is not critical at the filterbank stage, implying task-relevant information for each metric likely resides in similar frequency regions. Figure 11 in the Supplementary Materials further supports this, demonstrating that classifier choice had a more significant effect on frequency region distributions than the metric choice. The rSVM consistently outperformed rMDM in both cross-validation FB Optimisation and eventual SPDNet classification, with a larger performance gap in the CV setting.

rSVM and rMDM exhibited distinct frequency selection distributions. rSVM favoured more high-gamma regions, especially at low$N_{f}$, while rMDM generally showed lower frequency distributions, suggesting thinner band selection. As$N_{f}$increases, the filterbank’s effect becomes less discernible, particularly in the ChSpec setting. This trend may indicate the optimiser’s difficulty in handling numerous parameters (Frazier, 2018), leading to semi-random selections. This also explains the shape of the curves seen in Figure 11 of theSupplementary Materials, which are tending towards a random equal distribution of frequency regions (which would be expected to be a round curve centred at 124 Hz^§^). This would also explain the accuracy plateau observed at higher values of$N_{f}$.

#### 4.2.4 EEGSPDNet versus FBSPDNet

EEGSPDNet outperforms FBSPDNet in both the hyperparameter selection stage and then the final evaluation across multiple datasets, suggesting that learning the filterbank via backpropagation is preferable to optimisation in a separate stage. However, FBSPDNet offers immediate interpretability, through filterbank values and with FB-Opt CV scores potentially indicating final performance. FBSPDNet’s limitation lies in its inability to optimise in the entire convolutional space, precluding learning of complex filter types observed in Conv-EE(G)-SPDNet models.

#### 4.2.5 Network width

Network width, defined by the number of filters$N_{f}$(seeSection 2.5.2), was tested across all models. Increasing$N_{f}$generally improved classification ability, with the largest improvement typically observed between$N_{f}=1$and$N_{f}=2$. This performance boost could be attributed to increased network complexity and over-parameterisation, potentially allowing for “lucky” sub-networks at initialisation (Frankle & Carbin, 2019).

#### 4.2.6 Network depth

Network depth, defined by the number of BiMap-ReEig pairs ($N_{BiRe}$), was set to 3 for the proposed networks. An initial parameter sweep suggested$N_{BiRe}=3$as optimal, though this result should be interpreted cautiously due to the coarse nature of the sweep and the constant relative size of the following layer.

Only the first ReEig layer was found to be active in trained networks (Finding 6,Section 3.6). While this might suggest redundancy in latter ReEigs, it is important to note that this result is specific to the dataset and post-training state. Nonetheless, it is interesting to note that the majority of below-threshold eigenvalues were effectively “rectified” by the first BiMap layer.

TSMNet’s success with$N_{BiRe}=1$and a fixed size, made possible by its fixed output size spatial convolution layer, demonstrates an alternative viable method for constructing a DRN.

### 4.3 Insights from analyses

#### 4.3.1 Frequency analysis

In general, where peaks are observable, the assorted frequency domain plots (Figs. 5and6;Supplementary Figs. 10 and 11) show peaks at physiologically plausible points for the task of motor movement decoding. Namely, peaks can be observed at 10–20, 20–35, and 65–90 Hz (which roughly correspond to alpha, beta, and high-gamma regions) which agrees with previous literature regarding motor movement in EEG (Ball et al., 2008;Pfurtscheller & Neuper, 2001) and suggests that EE(G)-SPDNet is using known frequency bands for classification.

High gamma region utilisation varies across classifiers. For Conv-EE(G)-SPDNet, higher relative high gamma usage correlates with higher decoding performance, with ChSpec showing the highest usage, followed by ChInd and ChInd RmInt.

Conversely, Sinc-EE(G)-SPDNet exhibits an inverse trend in high gamma usage, with ChSpec using it least and the two ChInd models using it most.

In the ChSpec setting, high gamma usage decreases as$N_{f}$increases, while the opposite occurs for ChInd and ChInd RmInt. Despite this, the Sinc model sub-types follow the same decoding trend as the Conv model, with the highest accuracies achieved by the ChSpec variant.

#### 4.3.2 Architectural layer-by-layer analysis of EE(G)-SPDNet

Layer-by-layer performance analysis using Riemannian and Euclidean SVMs as proxy classifiers provided insights into information flow within the trained networks. Even though the decoding performance of the proxy classifier does not directly represent the network’s classification ability, this analysis reveals some interesting patterns.

The rSVM outperformed EE(G)-SPDNet in at least one layer for nearly every case, albeit by small margins (<1%). This shows the presence of task-relevant information inside the network that was not sufficiently extracted. Notably, when$N_{f}=1$, rSVM outperformed EEG-SPDNet by larger margins, with accuracies dropping most after the first BiMap-ReEig layer. This suggests information loss in the initial transformation, possibly due to under-parameterisation (i.e., the input covariance matrix is compressed too aggressively). This effect is reduced as the overall increase in network parameterisation (via increased$N_{F}$), supporting possible under-parameterisation.

The Euclidean SVM never outperformed SPDNet but showed interesting underperformance patterns. Channel-specific Conv-EE(G)-SPDNet performed best overall, requiring both channel specificity and Conv filtering. Uniquely, its final two layers often had the worst SVM accuracies for$N_{f}>2$, implying a distinct data transformation resulting in higher final accuracies but lower Euclidean space accuracies. How this final BiMap layer transformation relates to other results unique to the ChSpec Conv-EE(G)-SPDNet is still unclear.

### 4.4 Limitations

The results presented in this study are subject to several limitations. Computational constraints restricted the extent of hyperparameter space exploration, with the initial sweep of learning rates and weight decays being relatively coarse. Network width exploration was limited to$N_{f}=8$, though performance continued to increase without plateauing for EE(G)-SPDNet, suggesting potential for further improvements with wider networks. Furthermore, aside from an initial sweep, we did not explore network depth, changes to which could potentially yield decoding benefits. In particular, we also did not explore any variations in the BiMap reduction factor. Therefore, the assessment of the network decoding ability should be qualified by these limitations.

Additionally, the frequencies selected by learned filterbanks do not guarantee network utilisation of these frequencies. As filterbank width increases, either through higher$N_{f}$or ChSpec filtering, attributing decoding performance to individual filters becomes more challenging. This is also true for the multiband analysis: Manual inspection of the Electrode-Frequency relevance plots (Figs. 14and15) suggests their existence and potential importance for*some*models/participants, but this is not guaranteed. Furthermore, conclusions based off aggregated occurrences (Fig. 8) are limited by the detection method’s accuracy.

### 4.5 Future

In addition to the previously mentioned approaches such as NAS (Sukthanker et al., 2021), there are a number of other areas in which the ideas presented here could be improved. A comprehensive exploration of additional datasets, including other BCI datasets from MOABB (Jayaram & Barachant, 2018), EMG (Balasubramanian et al., 2018), and in-ear EEG (Yarici et al., 2022), would enable better model comparison and performance verification.

Methodological enhancements could include augmenting the covariance matrix with additional information such as done byWang et al. (2015), including a temporal covariance matrix (Hajinoroozi et al., 2017), using functional connectivity information (Chevallier et al., 2022), incorporating additional Riemannian layers, such as Riemannian Batch Norm (Brooks et al., 2019a;Kobler et al., 2022) (although this could be included as part of a NAS), spatial convolution layers (Kobler et al., 2022;Schirrmeister et al., 2017), and exploring learnable time-window selection (in a similar approach to optimised frequency band selection).

Further investigation of multiband filters is warranted and could involve refining the peak detection algorithm, developing filter relevance scores using gradient information, and creating a multiband sinc layer for SPDNet to compare with Sinc-EE(G)-SPDNet and Conv-EE(G)-SPDNet.

For FBSPDNet, there was no specific motivation for using a Bayesian optimiser over any other black-box optimiser, therefore exploring optimisers could yield improvements. Additionally, performance boosts could likely be achieved by exploring the optimisation hyperparameters or alternative proxy classifiers etc.

It would also be valuable to explore these methods on other EEG data types, such as steady-state visually evoked potentials (SSVEP) and the p300. In SSVEP-BCI systems (e.g.,Chevallier et al., 2018), users view multiple screens flickering at different frequencies. Neural activity is entrained to the flicker rate of the item attended to, with the trial labels corresponding to predetermined frequencies of the screens. Since the ideal frequency regions of interest are known*a priori*, expending computational resources to learn a filterbank*may*be unnecessary - depending on project goals.

For example, for anN-class SSVEP classification task, anNfilter ChInd Sinc-EE(G)-SPDNet could be constructed, where each of the sinc filters are fixed to the known frequency bands of the labels. Alternatively, for a more lightweight model, an$N_{F}=1$ChSpec Conv-EE(G)-SPDNet could be used. The decoding performance of this network would rely on the convolutional filters learning multiband filters to cover the range of labels and/or the channel specific filters filtering specific electrodes for each label.

The p300 is an event related potential (ERP) associated with sensory information processing that occurs roughly 300 ms after the onset of a stimulus (Picton, 1992). Since p300 information is primarily temporal rather than frequency-based, convolutional filters would likely outperform sinc filters as they could conceivably detect the p300’s temporal shape (or its p3a/p3b components). For this to be achieved however, the length of the convolutional kernel would have to be adjusted (in accordance with the sampling rate) such that it could encompass the whole p300 (or p3a/p3b). Whether the network learns some form of bandpass filter, a specialised p300/p3a/p3b filter, some hybrid of these two or something else entirely would be an interesting avenue to explore.

Finally, the hyperparameters of the networks could be explored further. As discussed in the limitations, higher values of$N_{f}$may yield further performance boosts. Additionally,$N_{BiRe}$could be further explored, especially in relation to the BiMap reduction factor. This could even be used to increase the size of the input SPD matrix, before compressing it later in the network. This could help capture the information that the LBL analysis suggests to have been poorly extracted in the early network.

## 5 Conclusion

In this paper, we have presented two novel Deep Riemannian Networks (DRNs) with learnable filterbanks (EE(G)-SPDNet & FBSPDNet) for decoding EEG BCI data, which were then shown to have state-of-the-art performance on public motor movement and motor imagery datasets. EEGSPDNet achieved the highest decoding accuracy among all proposed and comparison models, with statistically significant improvements over all but one competitor. These models have, therefore, shown improvement in what is arguably the most important factor of a BCI—raw decoding performance, while also providing a black-box approach to learning filterbank architecture. Standard filterbank architecture design is essentially a grid-search over the frequency band space, and the learned filterbank approach of FBSPDNet and EE(G)-SPDNet allows for an optimised search of the filter space. In particular, EE(G)-SPDNet, which encapsulates the filterbank optimisation as a convolutional layer, allows for this optimisation step to be completed enclosed within a single model, in an*end-to-end*manner. Furthermore, the convolutional layer was shown to be flexible and consistently selecting physiologically plausible frequency regions. Finally, the LBL analysis shows there is still room for improvement on the both of the proposed models, with a number of potential avenues of further exploration. In summary, through our strong decoding results, our exploration of learnable filterbank methodologies and hyperparameters, as well as our analysis into resulting network behaviour, we have established a foundational basis for end-to-end DRN design and analysis.

## Supplementary Material

Supplementary Material

## Data Availability

All datasets used in this study are publicly available, and were accessed via MOABB (Jayaram & Barachant, 2018). Code for the models can be found on the public GitHub repohttps://github.com/dcwil/eegspdnet.

## Footnotes

* The sample covariance matrix is the most common, but any other SPD matrix would be possible.

† It is also possible to flatten the input matrix entirely, but some early testing suggested keeping redundant information (from the off diagonals) at this stage decreased classification performance.

‡ SeeSupplementary Materialsfor proof.

§ This effect stems from the bandpass filter implementation. The optimiser adjusts a low-frequency cutoff and a bandwidth for each band. Cutoffs exceeding the Nyquist frequency are clamped before filter kernel computation. However, since the maximum bandwidth and maximum low cutoff are near the Nyquist frequency, the distribution of possible bands centres close to it.
