# Supplementary material for: Deep Riemannian Networks for end-to-end EEG decoding
Source: Imaging Neurosci (Camb). 2025 Mar 21;3:imag_a_00511. doi: 10.1162/imag_a_00511 (PMC12319850; doi:10.1162/imag_a_00511)
Supplement: Supplementary Material [file imag_a_00511-supp.pdf]

Supplementary Material: Deep Riemannian Networks for End-to-End EEG Decoding

Daniel Wilson*[1,2], Robin T. Schirrmeister[1,2], Lukas A. W. Gemein[1], Tonio Ball[1,2]

[1]Neuromedical A.I. Lab, Department of Neurosurgery, Medical Center - University of Freiburg,

Faculty of Medicine, University of Freiburg, Freiburg, Germany

[2]BrainLinks-BrainTools, IMBIT (Institute for Machine-Brain Interfacing Technology),

University of Freiburg, 79110 Freiburg im Breisgau, Germany

*Correspondence: daniel.wilson@uniklinik-freiburg.de

February 12, 2025

1 Supplementary Material

1.1 Proof for Equation 6

A definition for the definiteness of a matrix is for a real symmetric matrix, M:

$$\mathcal{S}_n := \{\, M = M^\top \mid m_{ij} \in \mathbb{R}^n\} \tag{1}$$

the scalar $z^\top M z$ will be positive (or non-negative in the case of positive semi-definite (PSD) matrices) for every non-zero column vector, $z \in \mathbb{R}^n$. Formally this is:

Let $M \in \mathcal{S}_n$. M is said to be positive-definite if:

$$z^\top M z > 0,\ \forall\ z \in \mathbb{R}^n - \{0\} \tag{2}$$

Let S_n and S_m be symmetric-positive definite (SPD) matrices of size n and m, respectively. Let

$Conc(S_n, S_m)$ be the mapping: $\mathcal{S}_n^{pd} \times \mathcal{S}_m^{pd} \Rightarrow \mathcal{S}_{n+m}^{pd}$ such that the output is a block diagonal matrix of the form:

$$C = Conc(S_n, S_m) = \begin{pmatrix} S_n & 0_{n\times m} \\ 0_{m\times n} & S_m \end{pmatrix} \tag{3}$$

Applying Equation 2 we get:

$$z^\top Cz = \begin{pmatrix} z_n^\top & z_m^\top \end{pmatrix} \begin{pmatrix} S_n & 0_{n\times m} \\ 0_{m\times n} & S_m \end{pmatrix} \begin{pmatrix} z_n \\ z_m \end{pmatrix} \tag{4}$$

which reduces to:

$$z_n^\top S_n z_n + z_m^\top S_m z_m \tag{5}$$

which will be > 0 provided S_n and S_m are positive definite, which was the initial assertion.

1.2 Frequency Gain Spectra

The order of calculations for frequency gain spectra is as follows: Data for a single model is of the form:

$$O = P \times S \times T \times E \times t_O \tag{6}$$

where P is the number of participants, S is the number of initialisation seeds for each network, T is the number of trials for each participant, E is the number of electrodes and t_O is the length of a trial in samples.

After going through the convolutional layer of a network the data array is of the form:

$$C = P \times S \times T \times Ch \times t_C \tag{7}$$

where Ch is the number of channels (E times number of filters, N_f) and t_C is the length of a trial after convolution.

Then the Fourier transform of the signals was calculated:

$$O \xrightarrow{FFT} \widetilde{O},\ C \xrightarrow{FFT} \widetilde{C} \tag{8}$$

With the length of the signals in $\widetilde{O}$ & $\widetilde{C}$ being reduced to $\widetilde{t}_O$ & $\widetilde{t}_C$, respectively.

Then the signals were averaged across trials:

$$\widetilde{O} \xrightarrow{AVG_T} \overline{\widetilde{O}} : P \times S \times E \times \widetilde{t}_O \tag{9}$$

$$\widetilde{C} \xrightarrow{AVG_T} \overline{\widetilde{C}} : P \times S \times Ch \times \widetilde{t}_C \tag{10}$$

$\overline{\widetilde{C}}$ was then interpolated (cubic) such that its signals were of the same length as $\overline{\widetilde{C}}$

$$\overline{\widetilde{C}} \xrightarrow{Interp} textcolorblackoverline\widetilde{C} : P \times S \times Ch \times \widetilde{t}_O \tag{11}$$

The electrode arrays in $\overline{\widetilde{O}}$ were then duplicated such that they matched the number of channels in $\overline{\widetilde{C}}$.

$$\overline{\widetilde{O}} : P \times S \times Ch \times \widetilde{t}_O \tag{12}$$

The signal gain (in dB), G, was then calculated:

$$G = 20log_{10}\left(\frac{\overline{\widetilde{O}}}{\overline{\overline{\widetilde{C}}}}\right) \tag{13}$$

1.3 Supplementary Comparisons

1.4 Additional LBL Figures

1.5 Additional Chosen Frequency Distributions

1.6 Software

All code was written with Python 3.8 and featured the following libraries:

- SciPy (Virtanen et al., 2020)
- NumPy (Harris et al., 2020)
- MNE (Gramfort et al., 2013)

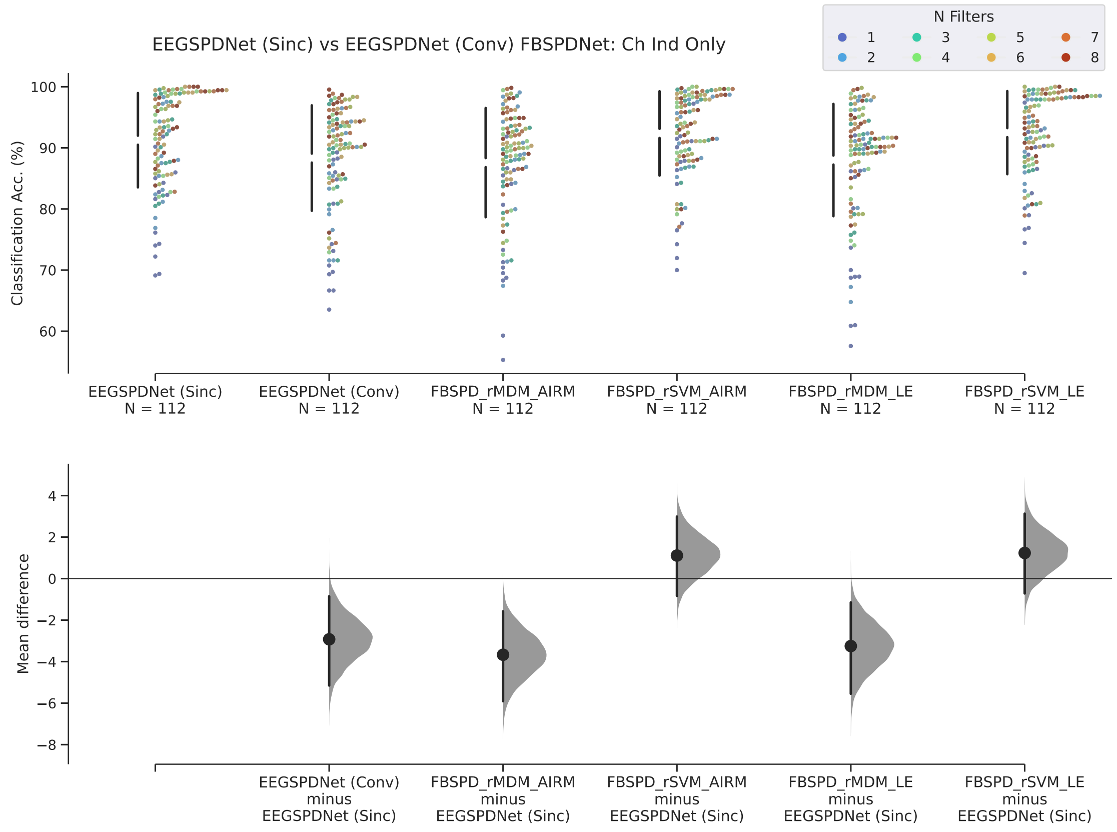

Figure 1: **Estimation Plot for Channel Independent Models.** The upper subplot is a swarm plot, with the models on x-axis, and the test-set classification accuracy on the y-axis. Each point represents a single participant training-testing loop for a given model. Hue denotes number of filters. To the left of a particular models swarm is the is a gapped line showing the swarm mean +/- standard deviation. The lower subplot shows the bootstrapped ($n = 10000$) mean difference between the left-most model (EEGSPDNet ChSpec) and every other model. The shaded area shows the distribution of the bootstrapped differences, with the dot and line respectively showing the mean an 95% confidence intervals. Here we show all ChInd EE(G)-SPDNet and FBSPDNet variants from the hyperparameter optimisation phase on Schirrmeister2017.

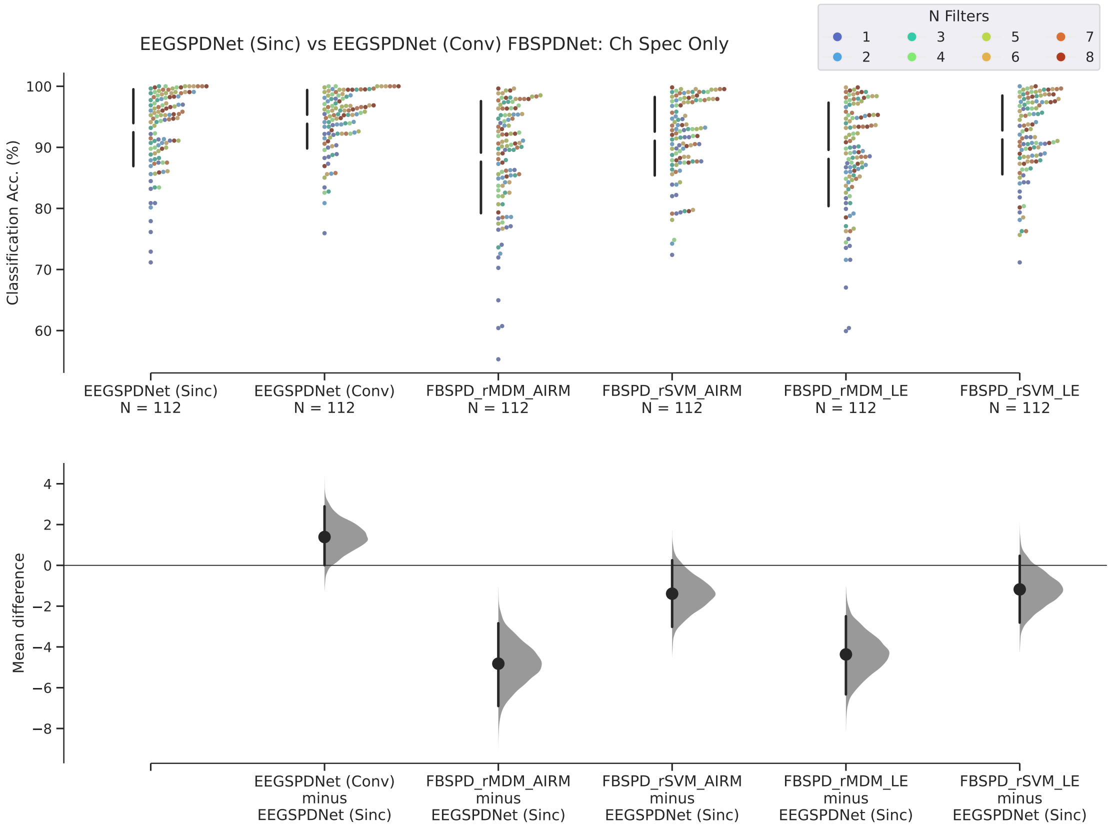

Figure 2: **Estimation Plot for Channel Specific Models.** A general description of estimation plot structure can be found in Figure 1. Here we show all ChSpec EE(G)-SPDNet and FBSPDNet variants from the hyperparameter optimisation phase on Schirrmeister2017.

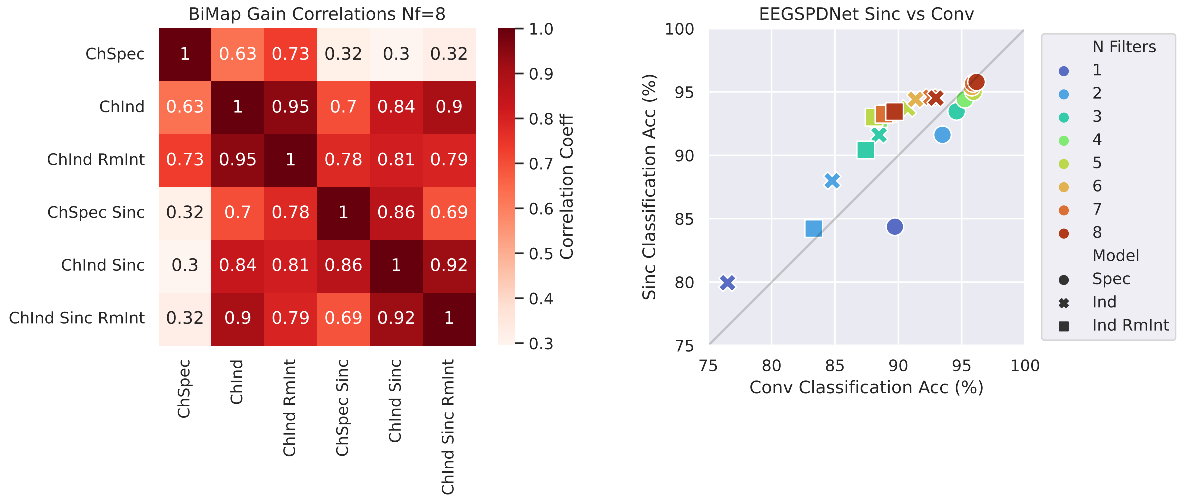

Figure 3: **BiMap Gain Correlations (left) and Classification Accuracy Scatterplot for Sinc and Conv EE(G)-SPDNet (right).** Left: Correlation coefficient matrix for spatial BiMap gain values seen in Figure 16 of the main text. Data highlights the similarity in spatial usage between ChInd and ChInd RmInt models and dissimilarity of ChSpec with many other models. Right: A general description of classification accuracy scatter plots can be found in the caption for Figure 4 of the main text.

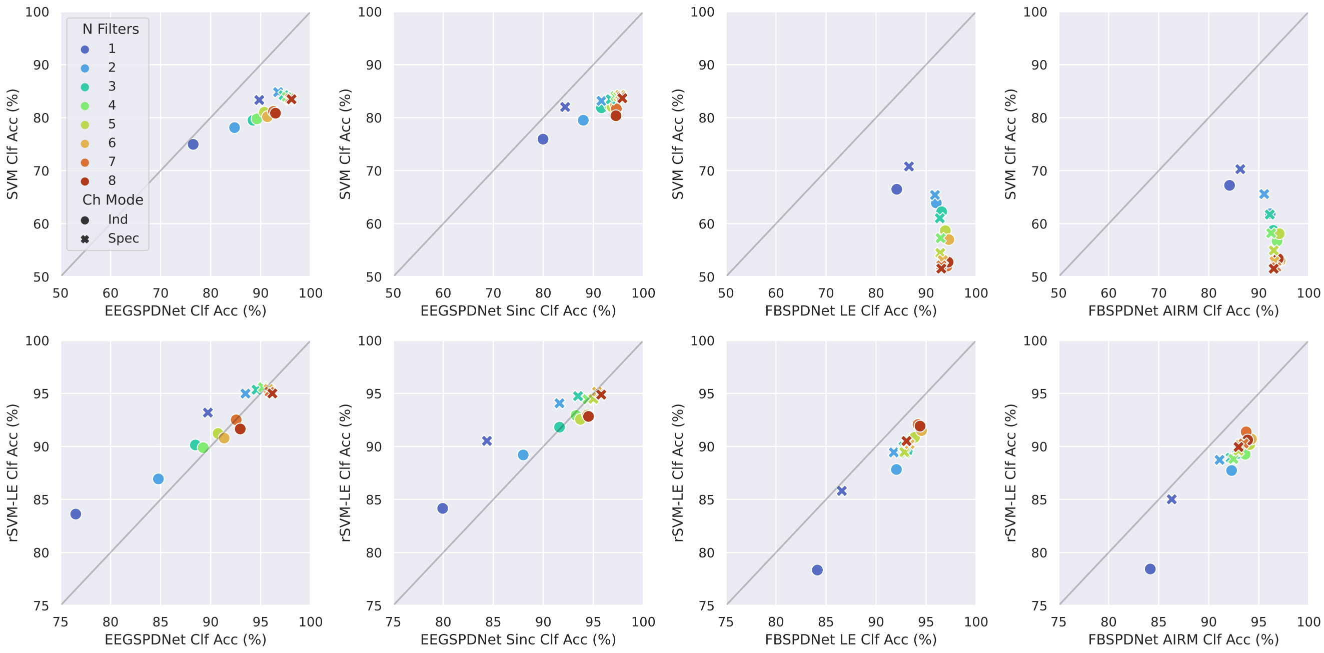

Figure 4: **SVM and rSVM-LE Accuracies on Post-Filterbank Covariance Matrices.** Y-axes show the SVM (top row) or rSVM (bottom row) classification accuracies. X axes show the EE(G)-SPDNet or FBSPDNet classification accuracies. Trials were filtered with the learned filterbank of the associated EE(G)-SPDNet or FBSPDNet. Note the different axes limits between the top and bottom rows. Hue and marker type respectively highlight N_f and channel specificity.

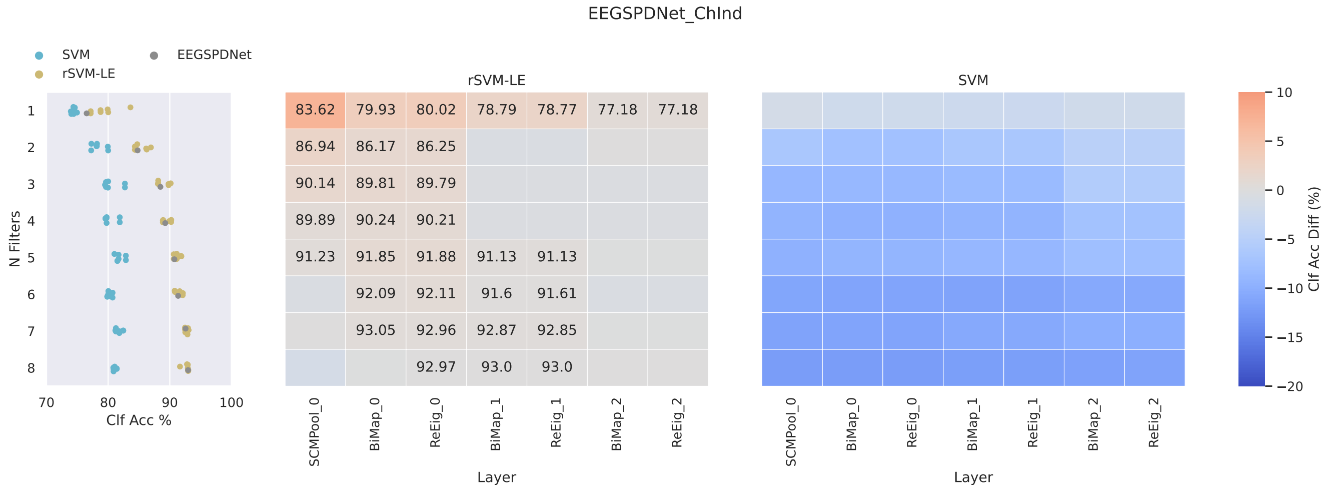

Figure 5: **LBL Performance of the Channel Independent EE(G)-SPDNet.** A general description of the LBL plots can be found in the caption for Figure 11 of the main text.

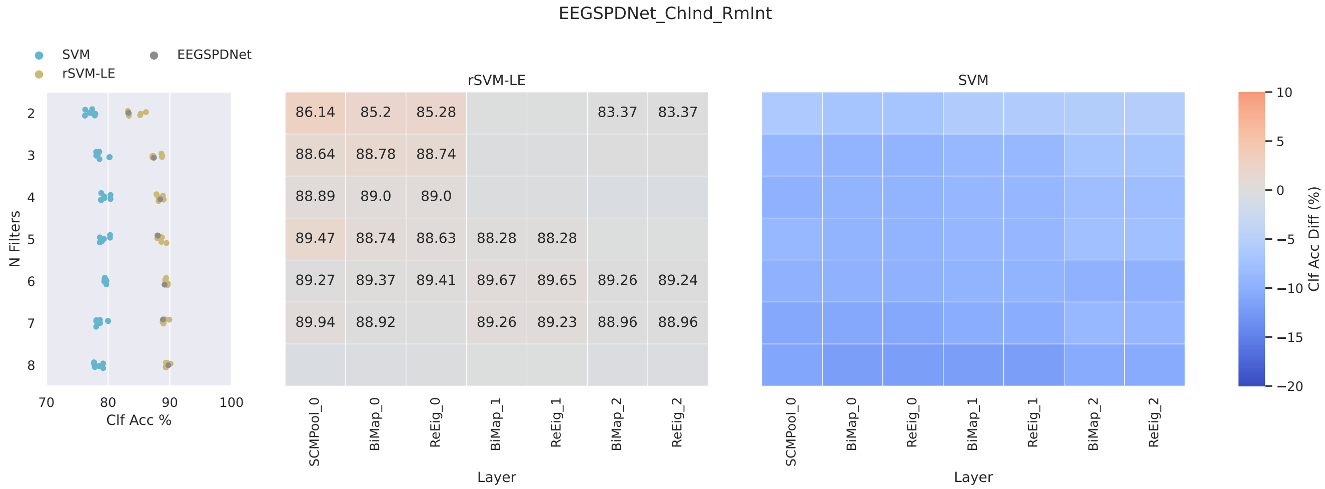

Figure 6: **LBL Performance for the Channel Independent EE(G)-SPDNet, without Interband Covariance.** A general description of the LBL plots can be found in the caption for Figure 11 of the main text.

- PyTorch (Paszke et al., 2019)
- Pandas (McKinney, 2010)
- Sci-Kit Learn (Pedregosa et al., 2011)
- Braindecode (Schirrmeister et al., 2017)
- Seaborn (Waskom, 2021)
- Matplotlib (Hunter, 2007)

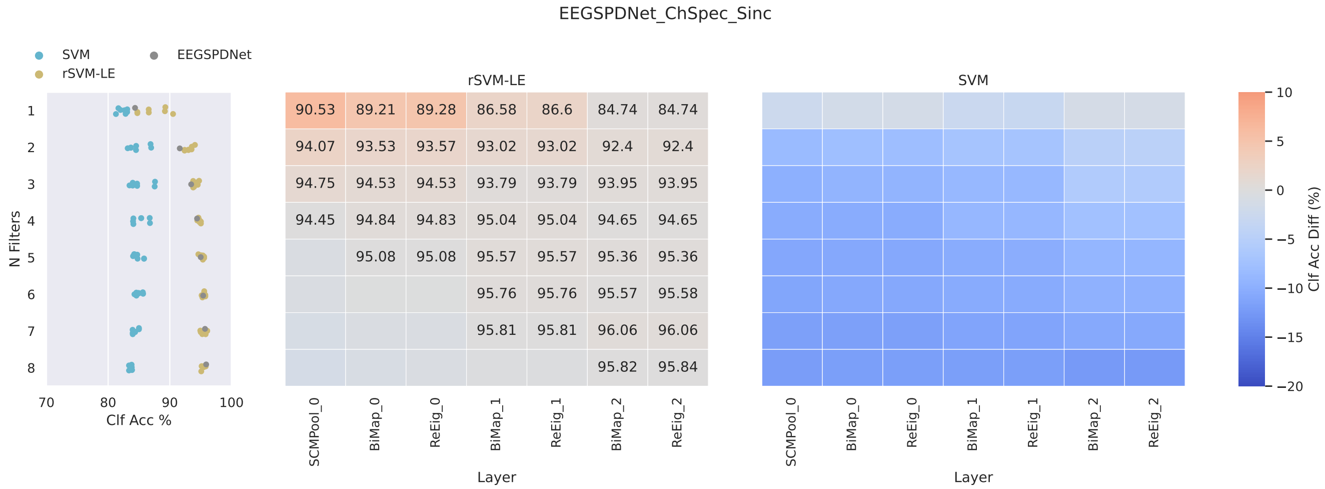

N Filters	SCMPool_0	BiMap_0	ReEig_0	BiMap_1	ReEig_1	BiMap_2	ReEig_2
1	90.53	89.21	89.28	86.58	86.6	84.74	84.74
2	94.07	93.53	93.57	93.02	93.02	92.4	92.4
3	94.75	94.53	94.53	93.79	93.79	93.95	93.95
4	94.45	94.84	94.83	95.04	95.04	94.65	94.65
5		95.08	95.08	95.57	95.57	95.36	95.36
6				95.76	95.76	95.57	95.58
7				95.81	95.81	96.06	96.06
8						95.82	95.84

Figure 7: **LBL Performance of the Channel Specific Sinc-EE(G)-SPDNet.** A general description of the LBL plots can be found in the caption for Figure 11 of the main text.

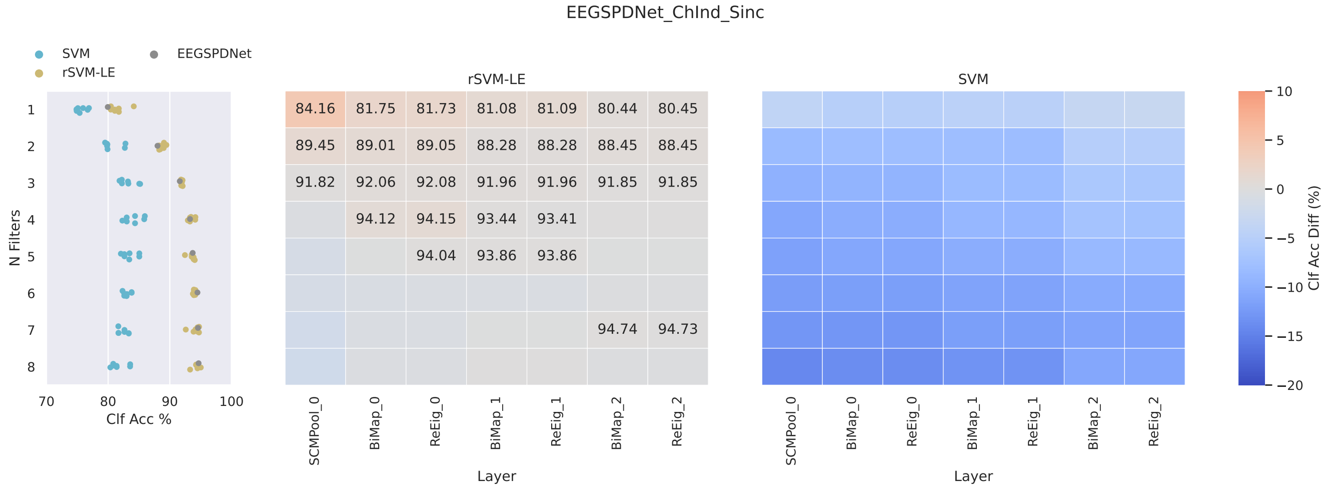

N Filters	SCMPool_0	BiMap_0	ReEig_0	BiMap_1	ReEig_1	BiMap_2	ReEig_2
1	84.16	81.75	81.73	81.08	81.09	80.44	80.45
2	89.45	89.01	89.05	88.28	88.28	88.45	88.45
3	91.82	92.06	92.08	91.96	91.96	91.85	91.85
4		94.12	94.15	93.44	93.41		
5			94.04	93.86	93.86		
6							
7						94.74	94.73
8							

Figure 8: **LBL Performance for the Channel Independent Sinc EE(G)-SPDNet.** A general description of the LBL plots can be found in the caption for Figure 11 of the main text.

- DabEst (Ho et al., 2019)
- bayes_opt (Nogueira, 2014–)
- geoopt (Kochurov et al., 2020)

Furthermore, we used and adapted code from the following libraries

- SPDNet (Python): https://github.com/adavoudi/spdnet
- TorchSPDNet (Python): https://gitlab.lip6.fr/schwander/torchspdnet/-/tree/master/

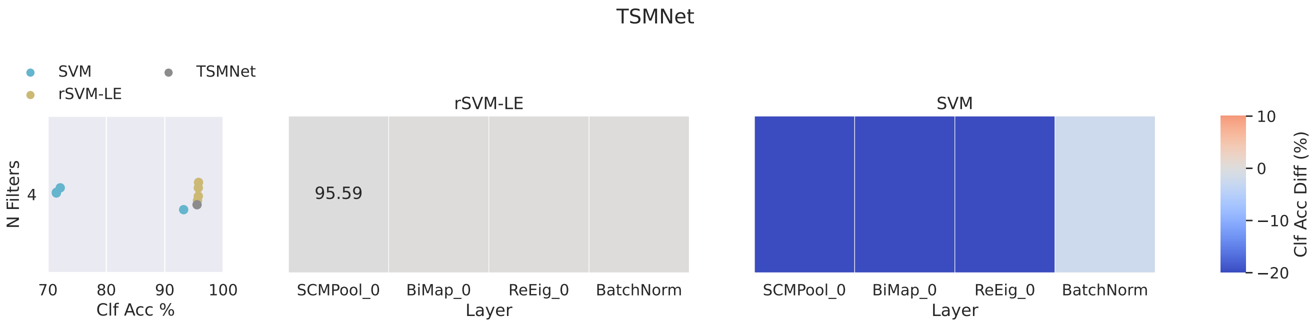

Figure 9: **LBL Performance for the TSMNet** A general description of the LBL plots can be found in the caption for Figure 11 of the main text.

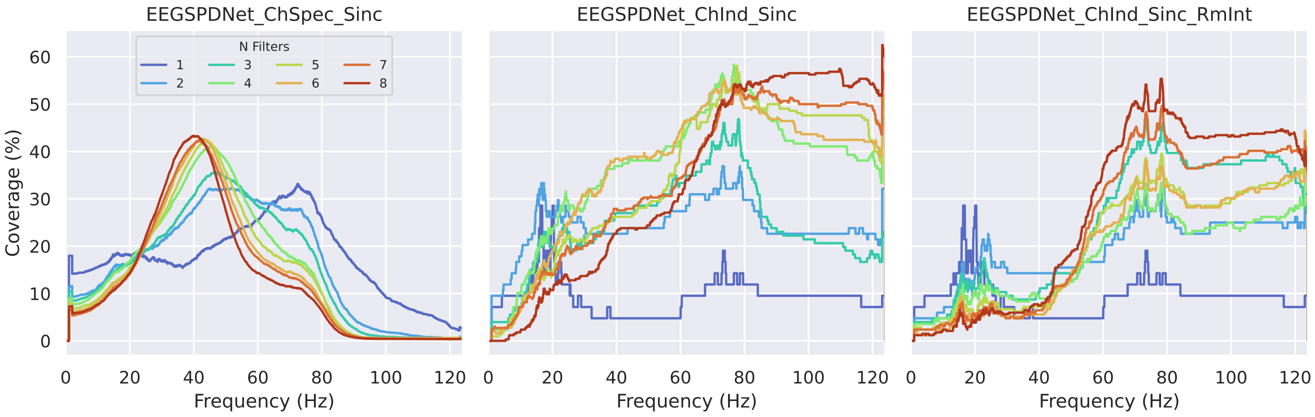

Figure 10: **Frequency Band Selection Distribution for Sinc-EE(G)-SPDNet.** A general description of the frequency band distribution plots can be found in the caption for Figure 7 of the main text.

- SPDNet (Matlab): https://github.com/zhiwu-huang/SPDNet
- TSMNet (Python): https://github.com/rkobler/TSMNet

Upon publication a GitHub repository containing the code used for generating the results will be made public.

1.7 Hardware

All computations were performed using bwForCluster NEMO, hardware details of which can be found here: https://wiki.bwhpc.de/e/NEMO/Hardware

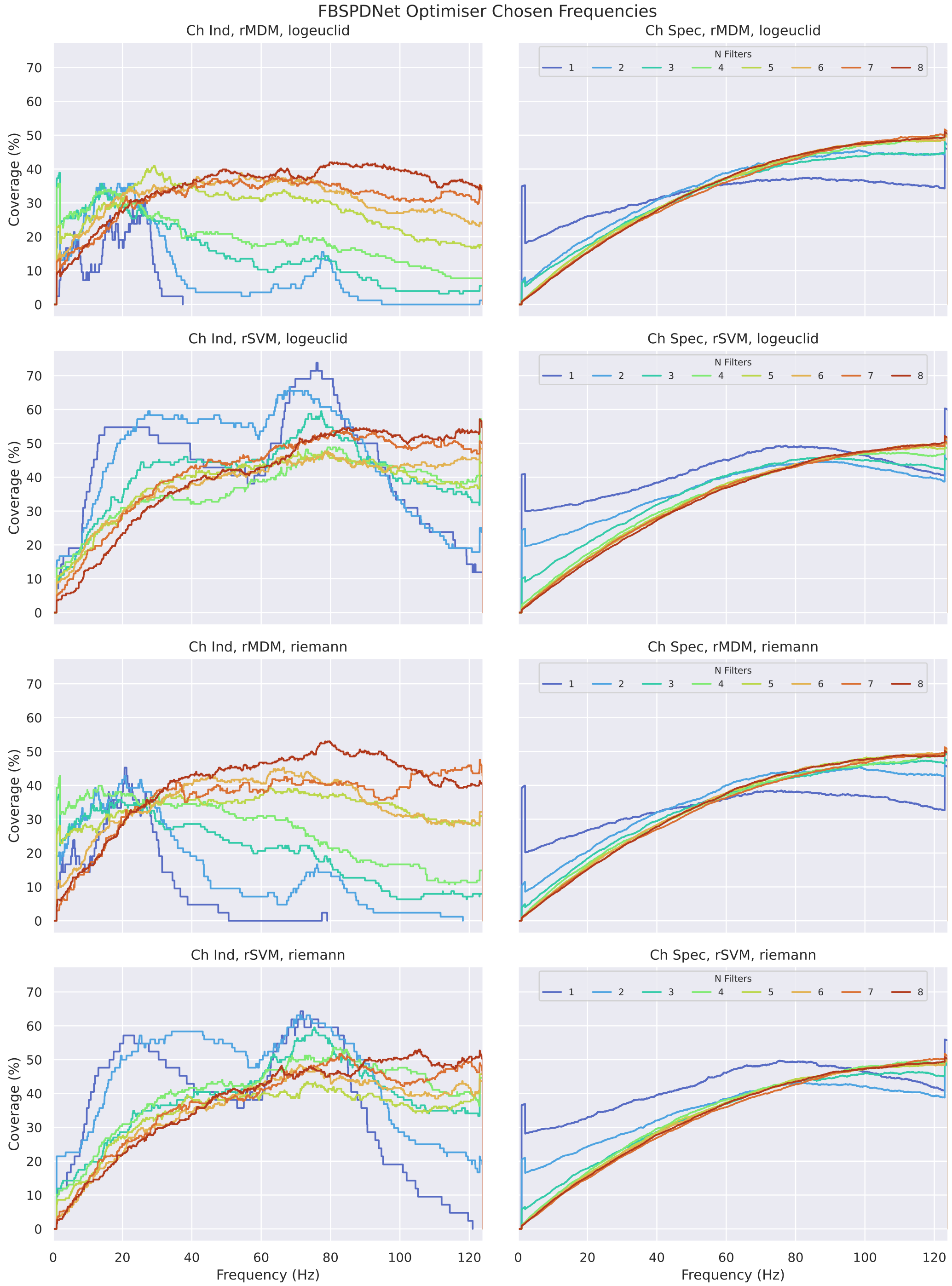

Figure 11: **Frequency Band Selection Distributions all FBSPDNet variants.** A general description of the frequency band distribution plots can be found in the caption for Figure 7 of the main text. Each subplot title respectively shows filter specificity, classifier, and Riemannian metric.

Table 1: **Dataset Information.** Table showing dataset metadata, more details can be found in the papers associated with each dataset (see Table), on the moabb website (http://moabb.neurotechx.com/docs/dataset_summary.html) and in the repository associated with this study.

	BNCI2014001[a]	BNCI2014004[b]	Lee2019 MI[c]	Schirrmeister2017[d]	Shin2017A[e]
# Electrodes	22	3	62	44	30
# Total Trials	5184	6520	11000	13440	1740
# Participants	9	9	54	14	29
# Classes	4	2	2	4	2
Samp Freq	250	250	250	250	200
Prepro Filtering	(4, 38)	(4, 38)	(4, 38)	(4, 124)	(4, 38)
Batch Size	120	120	80	256	20

[a] (Tangermann et al., 2012)
[b] (Leeb et al., 2007)
[c] (Lee et al., 2019)
[d] (Schirrmeister et al., 2017)
[e] (Shin et al., 2017)

1.8 Datasets

1.9 Parameter Sweeps

1.10 Model Hyperparameters

In this section the hyperparameters for the models can be found.

1.10.1 General

- Number of random seeds: 3
- SPD Estimator: Sample Covariance Matrix

1.10.2 SPDNet

- LR Scheduler: Cosine Annealing
- Epochs: 1000
- Loss: Cross-entropy loss
- ReEig Threshold: 5e-4

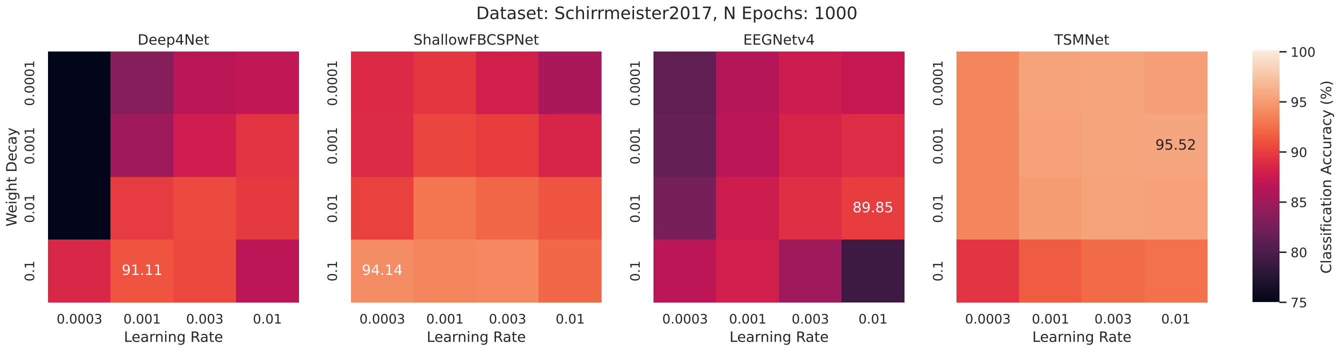

Figure 12: **Learning Rate & Weight Decay Parameter Sweep for Comparison Models.** Each heatmap subplot shows the coarse parameter sweep performed for selecting weight decay and learning rate for each model. The model is displayed as the heatmap title, see Section 2.8 in the main text for details on the comparison models. Weight decay values are shown on the y-axis, learning rate on the x-axis, with hue indicating the models classification performance on the test set (of the validation set, see Section 2.10 in the main text). The cell with the highest classification accuracy has been annotated.

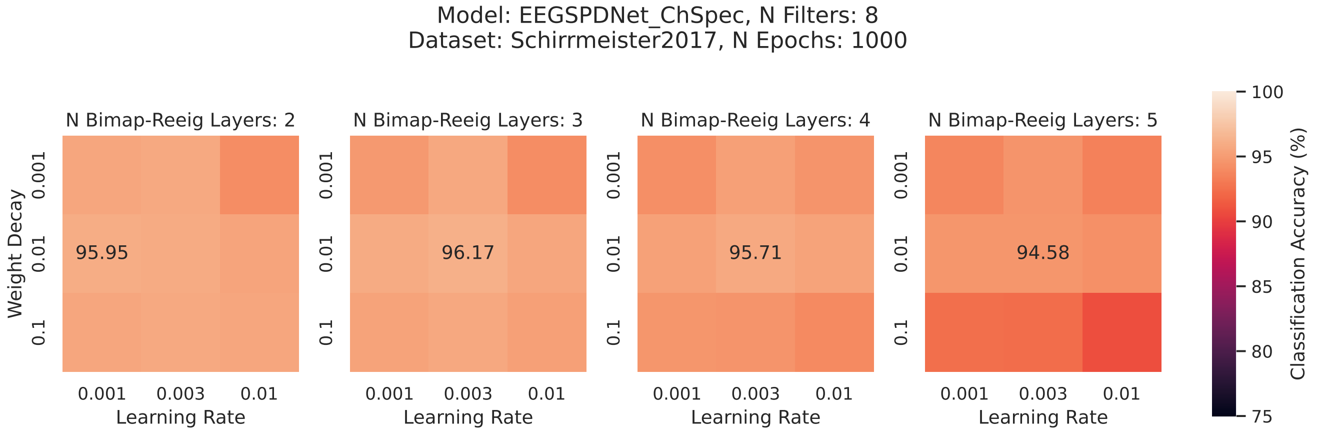

Figure 13: **Learning Rate & Weight Decay Parameter Sweep for EEGSPNet with Different** N_{BiRe}**.** See Figure 12 for figure structure details. Data shown is for EE(G)-SPDNet ChSpec, $N_f = 8$ and $N_{BiRe} \in \{2, 3, 4, 5\}$.

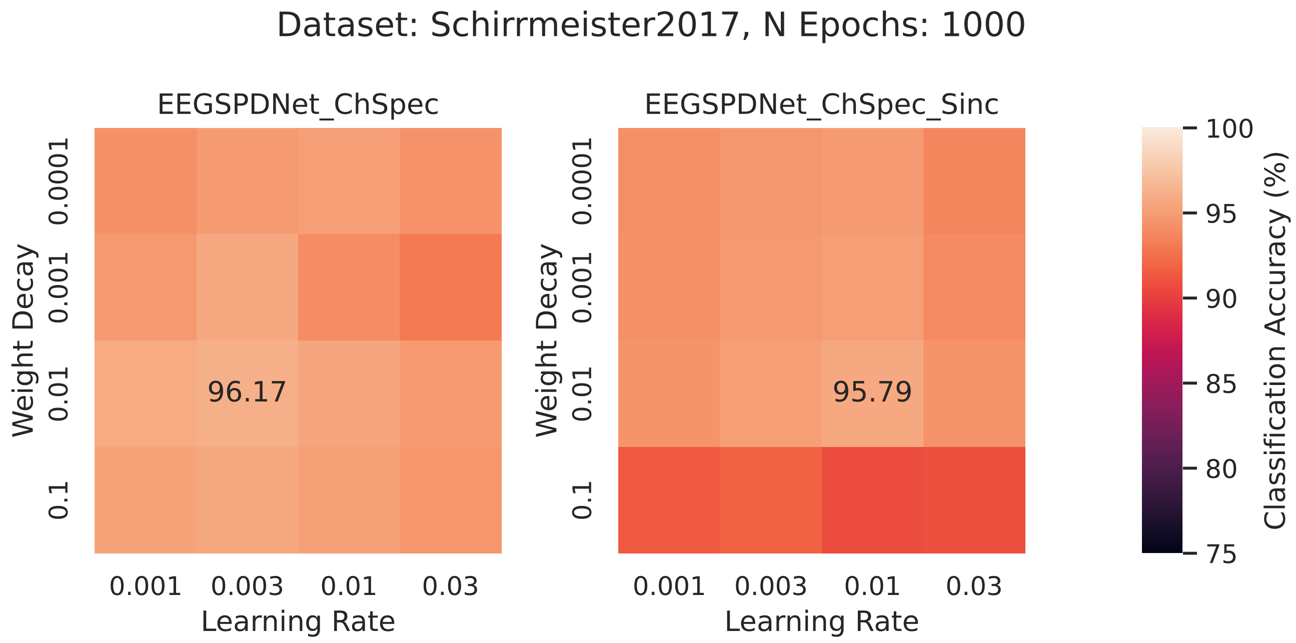

Figure 14: **Learning Rate & Weight Decay Parameter Sweep for EEGSPDNet** See Figure 12 for figure structure details. Data shown is for EE(G)-SPDNet ChSpec $N_f = 8$, for conv (left) and sinc (right) filtering.

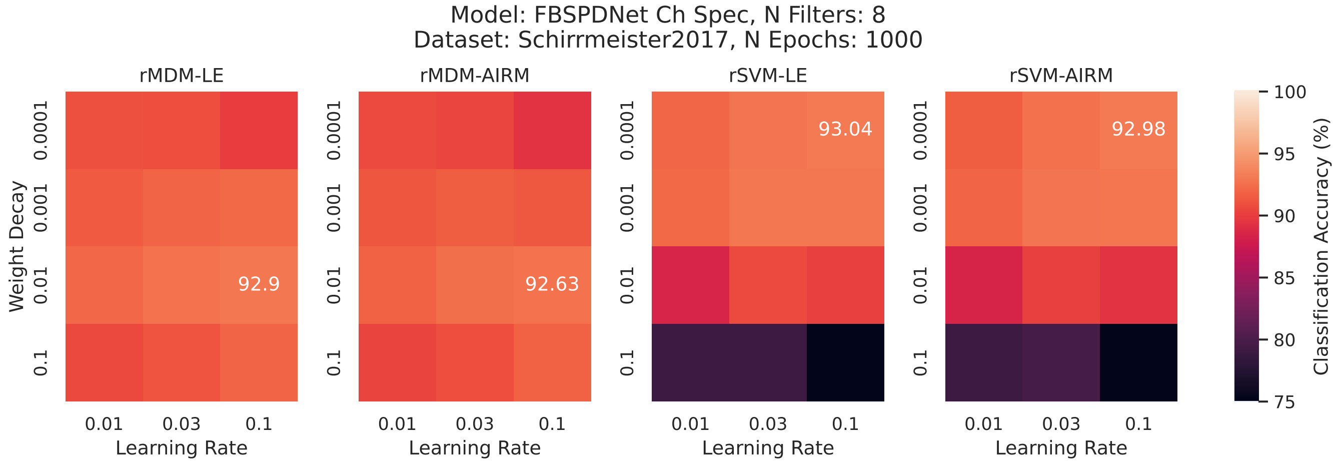

Figure 15: **Learning Rate & Weight Decay Parameter Sweep for FBSPDNet** See Figure 12 for figure structure details. Data shown is for FBSPDNet ChSpec, $N_f = 8$, for all the different proxy classifier configurations during filterbank optimisation. There are two classfiers, rMDM and rSVM, and two Riemannian metrics, LE and AIRM.

1.10.3 EE(G)-SPDNet

- Convolutional Kernel Length: 25

1.10.4 FBSPDNet

SVM parameters were chosen heuristically during initial testing.

- BO Stopping Criteria: 1000 iterations OR 12 hours
- SVM C: 1
- SVM Kernel: linear
- Cross-validation: Stratified 3-fold

1.11 Peak Detection

We used the "scipy.signal.find_peaks" function, with the following heuristically determined parameters. Other parameters were left at their default value.

- Peak Width: roughly 1Hz
- Peak Height: 1.5x Std Dev amplitude

References

Gramfort, A., Luessi, M., Larson, E., Engemann, D., Strohmeier, D., Brodbeck, C., Goj, R., Jas, M., Brooks, T., Parkkonen, L., & Hämäläinen, M. (2013). MEG and EEG data analysis with MNE-Python. *Frontiers in Neuroscience*, *7*. Retrieved January 30, 2023, from https://www.frontiersin.org/articles/10.3389/fnins.2013.00267

Harris, C. R., Millman, K. J., van der Walt, S. J., Gommers, R., Virtanen, P., Cournapeau, D., Wieser, E., Taylor, J., Berg, S., Smith, N. J., Kern, R., Picus, M., Hoyer, S., van Kerkwijk, M. H., Brett, M., Haldane, A., del Río, J. F., Wiebe, M., Peterson, P., ... Oliphant, T. E. (2020). Array programming with NumPy. *Nature*, *585*(7825), 357–362. https://doi.org/10.1038/s41586-020-2649-2

Ho, J., Tumkaya, T., Aryal, S., Choi, H., & Claridge-Chang, A. (2019). Moving beyond P values: Data analysis with estimation graphics [Publisher: Nature Publishing Group]. *Nature Methods*, *16*(7), 565–566. https://doi.org/10.1038/s41592-019-0470-3

Hunter, J. D. (2007). Matplotlib: A 2D Graphics Environment [Conference Name: Computing in Science & Engineering]. *Computing in Science & Engineering*, *9*(3), 90–95. https://doi.org/10.1109/MCSE.2007.55

Kochurov, M., Karimov, R., & Kozlukov, S. (2020, July). Geoopt: Riemannian Optimization in PyTorch [arXiv:2005.02819 [cs]]. Retrieved September 18, 2024, from http://arxiv.org/abs/2005.02819

Lee, M.-H., Kwon, O.-Y., Kim, Y.-J., Kim, H.-K., Lee, Y.-E., Williamson, J., Fazli, S., & Lee, S.-W. (2019). EEG dataset and OpenBMI toolbox for three BCI paradigms: An investigation into BCI illiteracy. *GigaScience*, *8*(5), giz002. https://doi.org/10.1093/gigascience/giz002

Leeb, R., Lee, F., Keinrath, C., Scherer, R., Bischof, H., & Pfurtscheller, G. (2007). Brain–Computer Communication: Motivation, Aim, and Impact of Exploring a Virtual Apartment. *IEEE Transactions on Neural Systems and Rehabilitation Engineering*, *15*(4), 473–482. https://doi.org/10.1109/TNSRE.2007.906956

McKinney, W. (2010). Data Structures for Statistical Computing in Python, 56–61. https://doi.org/10.25080/Majora-92bf1922-00a

Nogueira, F. (2014–). Bayesian Optimization: Open source constrained global optimization tool for Python. https://github.com/bayesian-optimization/BayesianOptimization

Paszke, A., Gross, S., Massa, F., Lerer, A., Bradbury, J., Chanan, G., Killeen, T., Lin, Z., Gimelshein, N., Antiga, L., Desmaison, A., Köpf, A., Yang, E., DeVito, Z., Raison, M., Tejani, A., Chilamkurthy, S., Steiner, B., Fang, L., ... Chintala, S. (2019, December). PyTorch: An Imperative Style, High-Performance Deep Learning Library [arXiv:1912.01703 [cs, stat]]. https://doi.org/10.48550/arXiv.1912.01703

Pedregosa, F., Varoquaux, G., Gramfort, A., Michel, V., Thirion, B., Grisel, O., Blondel, M., Prettenhofer, P., Weiss, R., Dubourg, V., Vanderplas, J., Passos, A., & Cournapeau, D. (2011). Scikit-learn: Machine Learning in Python. *Journal of Machine Learning Research*, *12*, 2825–2830.

Schirrmeister, R. T., Springenberg, J. T., Fiederer, L. D. J., Glasstetter, M., Eggensperger, K., Tangermann, M., Hutter, F., Burgard, W., & Ball, T. (2017). Deep learning with convolutional neural networks for EEG decoding and visualization. *Human Brain Mapping*, *38*(11), 5391–5420. https://doi.org/https://doi.org/10.1002/hbm.23730

Shin, J., von Lühmann, A., Blankertz, B., Kim, D.-W., Jeong, J., Hwang, h.-j., & Müller, K.-R. (2017). Open Access Dataset for EEG+NIRS Single-Trial Classification. *IEEE Transactions on Neural Systems and Rehabilitation Engineering*, *25*, 1735–1745. https://doi.org/10.1109/TNSRE.2016.2628057

Tangermann, M., Müller, K.-R., Aertsen, A., Birbaumer, N., Braun, C., Brunner, C., Leeb, R., Mehring, C., Miller, K. J., Müller-Putz, G. R., Nolte, G., Pfurtscheller, G., Preissl, H., Schalk, G., Schlögl, A., Vidaurre, C., Waldert, S., & Blankertz, B. (2012). Review of the BCI Competition IV. *Frontiers in Neuroscience*, *6*, 55. https://doi.org/10.3389/fnins.2012.00055

Virtanen, P., Gommers, R., Oliphant, T. E., Haberland, M., Reddy, T., Cournapeau, D., Burovski, E., Peterson, P., Weckesser, W., Bright, J., van der Walt, S. J., Brett, M., Wilson, J., Millman, K. J., Mayorov, N., Nelson, A. R. J., Jones, E., Kern, R., Larson, E., . . . van Mulbregt, P. (2020). SciPy 1.0: Fundamental algorithms for scientific computing in Python [Number: 3 Publisher: Nature Publishing Group]. *Nature Methods*, *17*(3), 261–272. https://doi.org/10.1038/s41592-019-0686-2

Waskom, M. L. (2021). Seaborn: Statistical data visualization. *Journal of Open Source Software*, *6*(60), 3021. https://doi.org/10.21105/joss.03021
